# Biomechanical Simulation of Stress Concentration and Intraocular Pressure in Corneas Subjected to Myopic Refractive Surgical Procedures

**DOI:** 10.1038/s41598-017-14293-0

**Published:** 2017-10-24

**Authors:** Po-Jen Shih, I-Jong Wang, Wen-Feng Cai, Jia-Yush Yen

**Affiliations:** 1 Department of Civil and Environmental Engineering, National University of Kaohsiung, 81148 Kaohsiung, Taiwan; 2 Department of Ophthalmology, College of Medicine, National Taiwan University, 10048 Taipei, Taiwan; 3 Department of Mechanical Engineering, National Taiwan University, 10617 Taipei, Taiwan

## Abstract

Recent advances in the analysis of corneal biomechanical properties remain difficult to predict the structural stability before and after refractive surgery. In this regard, we applied the finite element method (FEM) to determine the roles of the Bowman’s membrane, stroma, and Descemet’s membrane in the hoop stresses of cornea, under tension (physiological) and bending (nonphysiological), for patients who undergo radial keratotomy (RK), photorefractive keratectomy (PRK), laser-assisted *in situ* keratomileusis (LASIK), or small incision lenticule extraction (SMILE). The stress concentration maps, potential creak zones, and potential errors in intraocular pressure (IOP) measurements were further determined. Our results confirmed that the Bowman’s membrane and Descemet’s membrane accounted for 20% of the bending rigidity of the cornea, and became the force pair dominating the bending behaviour of the cornea, the high stress in the distribution map, and a stretch to avoid structural failure. In addition, PRK broke the central linking of hoop stresses and concentrated stress on the edge of the Bowman’s membrane around ablation, which posed considerable risk of potential creaks. Compared with SMILE, LASIK had a higher risk of developing creaks around the ablation in the stroma layer. Our FEM models also predicted the postoperative IOPs precisely in a conditional manner.

## Introduction

From the viewpoint of biomechanics and safety, corneas undergoing refractive surgery must maintain a stable shape and avoid postsurgical biomechanical decompensation, which may result in an unsatisfactory visual recovery and complications^1,2^. The progress of refractive surgery from radial keratotomy (RK) using a diamond knife^3^ to automated lamellar keratoplasty using a mircrokeratome, photorefractive keratectomy (PRK), laser-assisted subepithelial keratectomy (LASEK), laser-assisted *in situ* keratomileusis (LASIK), refractive lenticule extraction, and small incision lenticule extraction (SMILE) using excimer lasers or femtosecond lasers has increased the precision and safety of the method^1^. In addition, the increasing knowledge of corneal biomechanics has contributed to both promising results and increasing postsurgical safety. However, iatrogenic corneal ectasia remains the most feared scenario that can occur after an uneventful corneal laser surgery, although a reduction in its incidence from 4% to 2.8% has been reported following LASIK, PRK, or other corneal refractive procedures^4^. Risk factors for iatrogenic corneal ectasia include thin corneas (<500 µm), a pathological corneal topography, a residual stromal bed of <300 µm, high myopia of <−8 D, age of <25 years (in females), atopic dermatitis, allergies, family history for keratoconus and collagen diseases, and retreatments^5^. In severe cases, a penetrating keratoplasty or a deep anterior lamellar graft is necessary^6^.

For safety, ideal refractive surgery aims to maintain a stable response of central flattening and peripheral steepening in a stiff cornea preoperatively and postoperatively^7^. Therefore, corneal biomechanics must be measured correctly to prevent adverse events after surgery. Currently, two devices are available for measuring some biomechanical properties and providing a clinical assessment of corneal deformation response, namely the Corvis^®^ ST (Oculus Inc., Berlin, Germany) and ocular response analyser (ORA; Reichert Inc., Depew, NY). However, both these devices cannot accurately predict the possibility of iatrogenic ectasia before surgery^7,8^. Furthermore, modern laser refractive surgical procedures alter the corneal lamellae or collagen fibres by laser ablation or disruption. These procedures differ from the incisions in corneas made during RK; thus, different responses of corneal biomechanics result from modern laser refractive surgical procedures. Accordingly, complaints of glare, halo effect, fluctuating vision, corneal fragility during trauma, and a progressive hyperopic change might differ among these surgical procedures when assessed in a long-term follow-up after surgery^9^. A preoperative study of corneal biomechanics is necessary for preventing complications and iatrogenic ectasia. Furthermore, the biomechanical parameters of the cornea include a function of the intraocular pressure (IOP) and the stiffness of the sclera^10^ because IOP measurements with applanation tonometry are not valid once the corneal structure is altered^7,11^. Furthermore, patient-specific numerical determination^12^ is a crucial safety concern for patients who seek surgery. Ariza-Gracia *et al*. reported that the noncontact tonometry test is not sufficient to evaluate the individual mechanical properties and a complete *in vivo* characterization of the preoperative cornea, in which the anterior cornea presents compression in a range of 0–0.4 MPa individually^13^. Other *ex vivo* measurements with a numerical-experimental protocol, which include biaxial tension and bending experiments, should be used to calibrate corneal mechanical properties individually^14,15^. Furthermore, measuring the entire shape of the cornea^16,17^ instead of the apical displacements, with an *in vivo* biomechanical analyser is indispensable for this purpose^18^, because the limbus stiffness is 47.3% lower than that of the central cornea.

In current study, we applied the finite element method^13,19,20^ (FEM) and the Goldmann applanation principle^21^ to simulate the biomechanical responses in corneal layer structures having undergone RK, PRK, LASIK^22,23^, and SMILE^24^. The material behaviours of corneas were regarded as nearly incompressible anisotropic hyperelastic behaviours, as described in previous constitutive models^23,25–27^, and the biomechanical behaviours of corneas that underwent refractive surgeries were authenticated through considerations of geometry^28,29^, boundary conditions^20,30^, and mesh analysis^13,31,32^. The simulated corneal deformation and created stress maps^33,34^, when the corneas were subjected to the influence of IOP and applanation pressure (IOP_a_), were based on the basis of previously reported Young’s moduli of layered corneal structures^28^. Finally, the analyses of stresses and displacements were compared with previous simulations^21,24,35,36^ and experiments^37–39^. Our results indicated that the major hoop stress acts on the circumference and parallel to the length of the cornea, which results in tensile stress in the meridian directions. Different potential creak zones were noted in the layers of corneas that had undergone refractive surgical procedures and had been subjected to eye rubbing conditions.

## Results

### Corneal shape changes and hoop stress distribution

**Figure 1:**
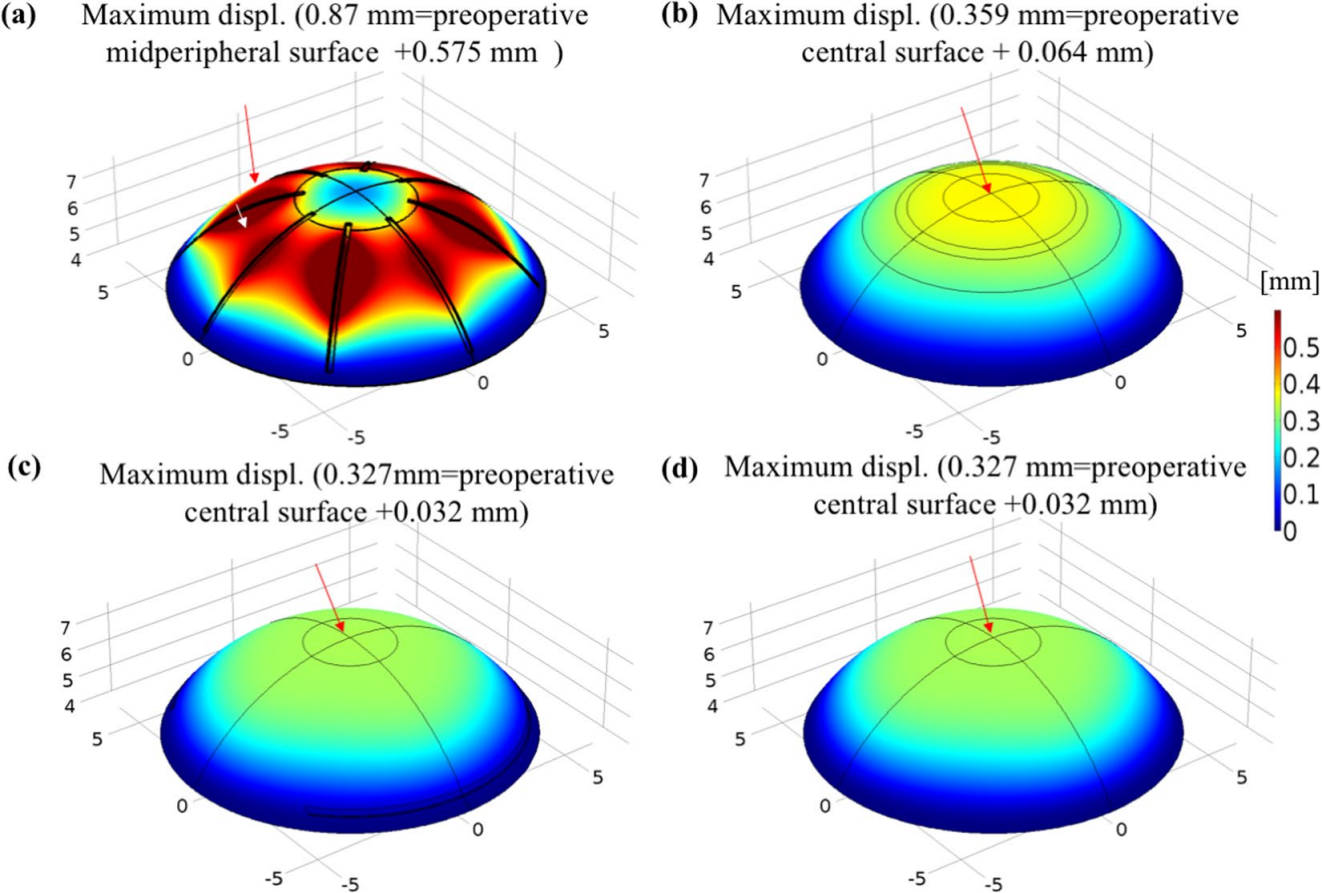
Simulated displacements in corneal shape on the surface resulting from the four refractive surgical procedures at a normal IOP of 15 mmHg. The dark-red areas involve maximum displacements (>0.5 mm) outwards (body expansion), and the dark-blue areas involve zero displacement near the constrained boundary of the models. The “preoperative surface” is displacement of the normal cornea (from Fig. 10a). (**a**) RK: maximum displacements located at middle incisions; (**b**) PRK: maximum displacement at central cornea; and (**c**) LASIK and (**d**) SMILE: maximum displacements located around the central cornea (Unit: mm).

**Figure 2:**
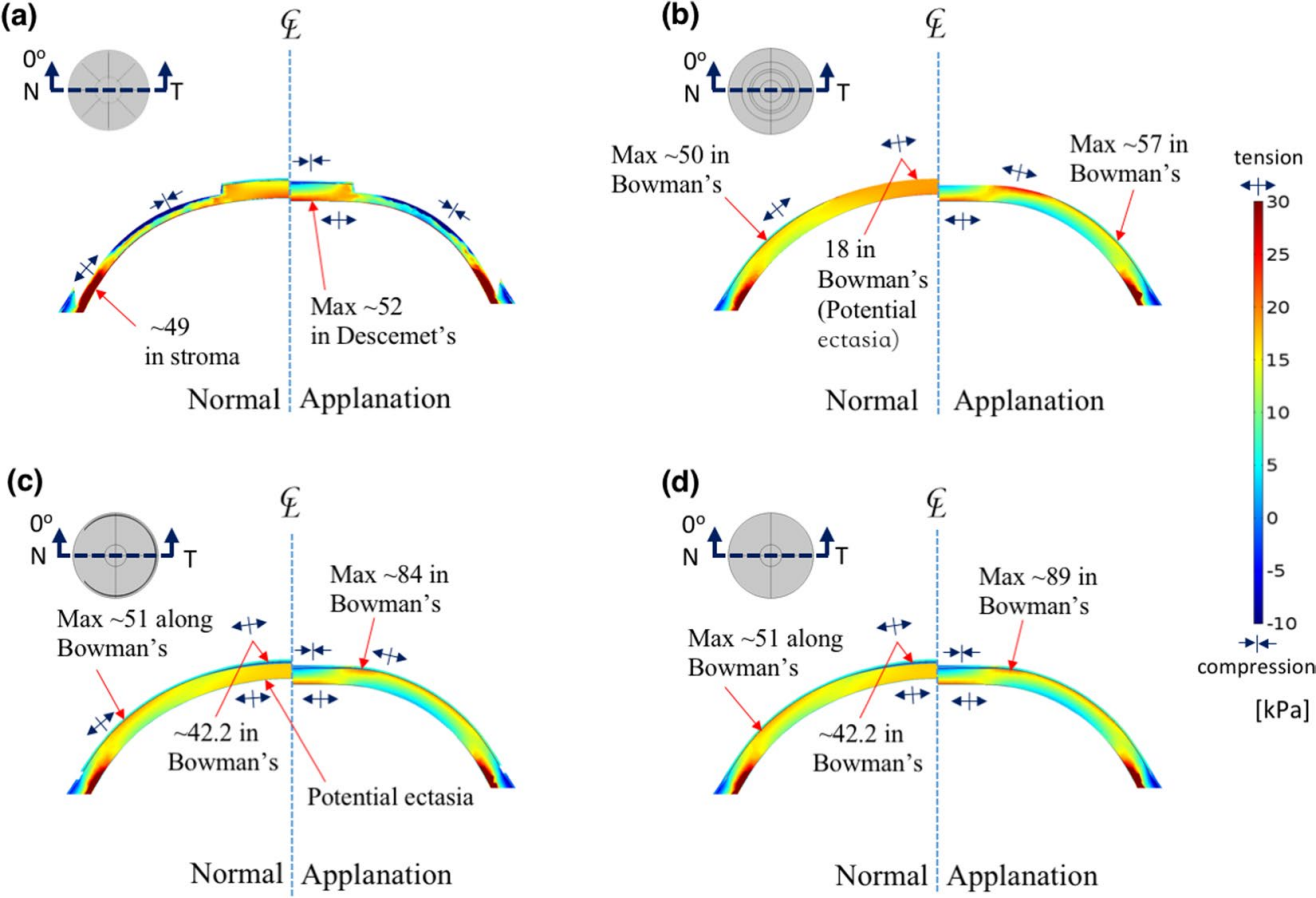
Hoop stress distribution, ${{\rm{\sigma }}}_{{\rm{\varphi }}}$, represents circumferential tension in the tangential direction along the cornea, on the N–T slices of the corneas of the four models under normal (left) and applanation (right) conditions. (**a**) RK: the zone with the negative stress is the compression zone located around the middle of the incision; (**b**) PRK: the highest tension is near the edge of the ablation and the central bottom area; (**c**) LASIK: the highest stresses are near the edge of ablation (the compression zone is found on the top surface of the ablation, and the zone with the highest tension is near the ablation edge); (**d**) SMILE: the potential ectasia zone is the same as that of LASIK (value of the legend shows tension; unit: kPa).

Based on our FEM models, the simulations of the changes of corneal shape and hoop stress distribution associated with the four refractive surgical procedures (5D) are illustrated in Figs 1 and 2. For RK, because of the combined effects of IOP and the changes in corneal integrity, the midperipheral cornea was deformed outwardly (575 µm elevation from the surface of the preoperative cornea) to a greater extent than were other parts of the cornea, as illustrated in Fig. 1a; however, the central cornea showed less displacement. For PRK, because the superficially central stroma and Bowman’s membrane were ablated, the inner pressure pushed the central cornea outwards, as shown in Fig. 1b. The deformation patterns observed from LASIK and SMILE models (Fig. 1b and c) were similar to those of PRK (Fig. 1b), and the maximum displacement occurred at the central cornea.

Figures 2 and S1 presents the hoop stress, ${\sigma }_{\varphi }$, on the cornea under normal (left) and applanation (right) conditions; the four refractive surgery models with 5D correction are also demonstrated for further analysis. The colour scales in Fig. 2 are identical in these four models. The hoop stress acted on the circumference and parallel to the length of the cornea, describing the tension in the meridian ($\varphi$) direction. Since collagen lamellae in the stroma appear to be close to each other in the inferior–superior (I–S) and nasal–temporal (N–T) directions^40–43^, the incisions of the RK would loosen or break these fibres (−90%). The negative ${\sigma }_{\varphi }$ (compression) in Fig. 2a is indicated in the major axis of the midperipheral cornea but positive ${\sigma }_{\varphi }$ (tension) is indicated in the minor axes in Figure S1a and e. For the PRK model, the apical stress ${\sigma }_{\varphi }$ was 18 kPa, which was slightly less than the 19.62–21.57 kPa stress range from the other simulation model^38^. The highest ${\sigma }_{\varphi }$ was located in the central cornea without applanation, and the area of highest stress was located around the ablated edge under applanation, as depicted in Figs 2b and S1b. For the LASIK model, the highest ${\sigma }_{\varphi }$ was located in the stroma and in the Bowman’s membrane around the ablated edge, as shown in Figs 2c and S1c. For the SMILE model shown in Fig. 2d, the apical maximum stress ${\sigma }_{\varphi }$ was 42.2 kPa, which was higher than the 35 kPa predicted by the simulation model of Studer *et al*.^24^. The highest ${\sigma }_{\varphi }$ was also located near the ablated edge, and was higher than the stress in the LASIK model, which is compatible with the findings of Roy *et al*.^35^. We reason that the flap edge of LASIK interrupts the stress in the Bowman’s membrane and decreases the stress in the midperipheral cornea. Thus, the stress is transferred to the minor axis and increases the values as illustrated in Figure S1c. Upon applanation, the hoop stress, ${\sigma }_{\varphi }$, in the central cornea decreased because the applanation force bent the central cornea inward, thus compressing the top of the central cornea^14^.

### First principal stress (FPS) analysis

**Figure 3:**
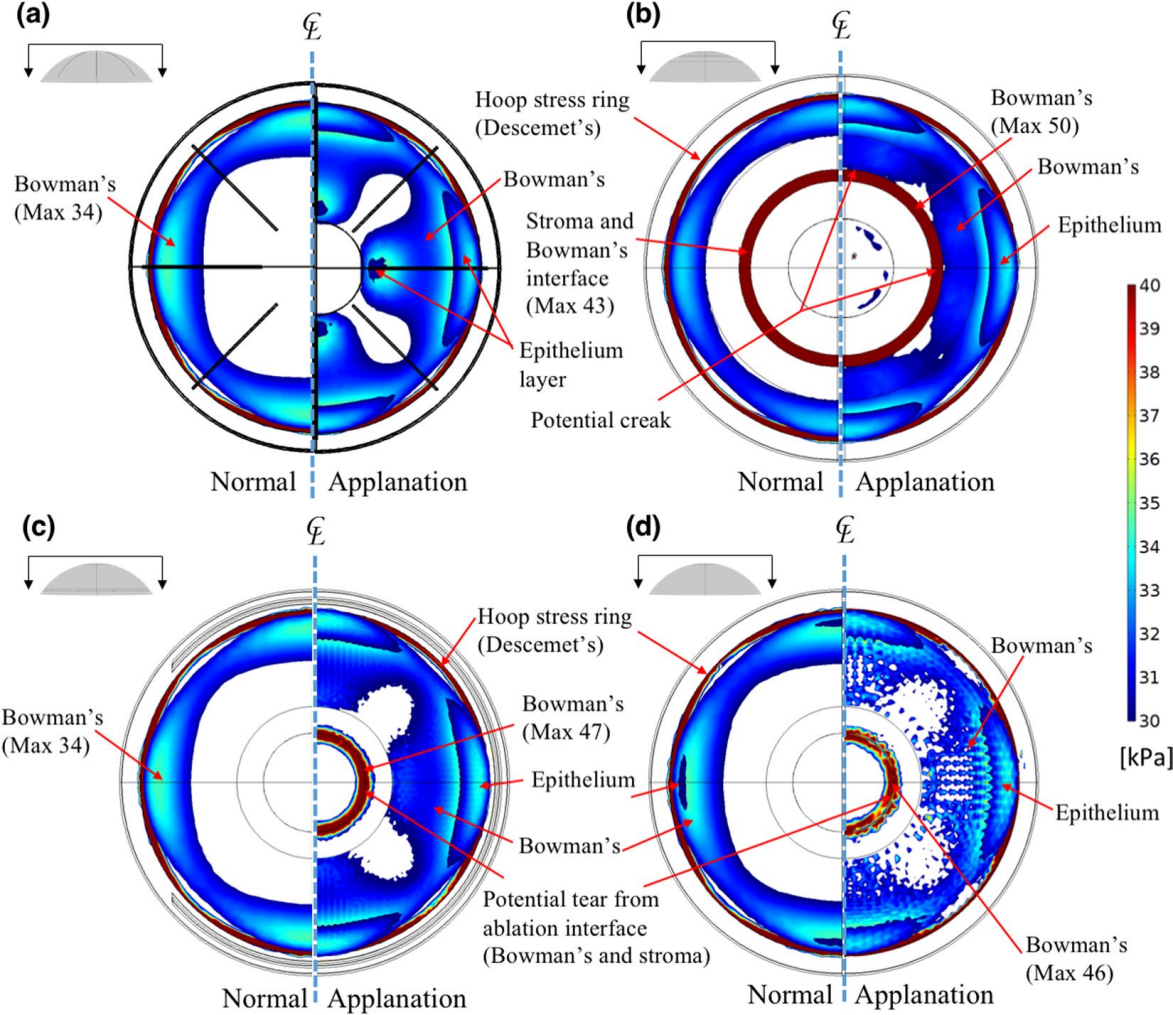
High FPS areas (top view showing FPS >30 kPa) of the four models without (left) and with (right) applanation conditions. (**a**) RK: the zones with the highest stress are associated with the directions of fibres and incisions; (**b**) PRK: the potential creak zone is near the edges of ablation; (**c**) LASIK: the potential creak zone is near the edge of ablation and 45° cracks in radial directions; (**d**) SMILE: the potential creak zone is the same as that of LASIK but the maximum stress is less than that in LASIK (unit: kPa).

The FPS analysis of these four models facilitated the investigation of the maximum stresses in corneas subjected to refractive surgical procedures with or without applanation. The FPS is the component of the stress tensors when the basis is rotated to reduce the shear stress component to zero^44^. Once the material properties of the layered cornea, particularly the yielding stress and broken stress, are provided, potential damage under high FPS conditions can be predicted as expected. Thus, the cornea under tension might tear perpendicularly to the FPS direction. Accordingly, Figs 3 and S2 depict the stress distribution associated with a high FPS under normal conditions (left) and under the applanation condition (right). The colour scales in Fig. 3 are the same and indicate FPS values exceeding 30 kPa. The PRK model was observed to have a higher FPS than the other models (i.e., FPS = 50 kPa). In the RK model, the potential creak zones may be near the incisions of the Bowman’s membrane as shown in Figures S2a. For the PRK model, potential creaks may be located near the ablated zone, as shown in Fig. 3b. For the LASIK and SMILE models, the high FPS was located in the centre of the ablated zone in the Bowman’s membrane, as shown in Figs 3c,d,S2c, and S2d. Notably, the area of stress distribution of the LASIK model was larger than that of the SMILE model because LASIK involved the creation of a corneal flap. Moreover, the RK stress pattern resembled the LASIK stress pattern because the incisions that were parallel to I–S and N–T directions had little influence on breaking stress and the incisions at 45° exerted effects on the small stress zones.

**Figure 4:**
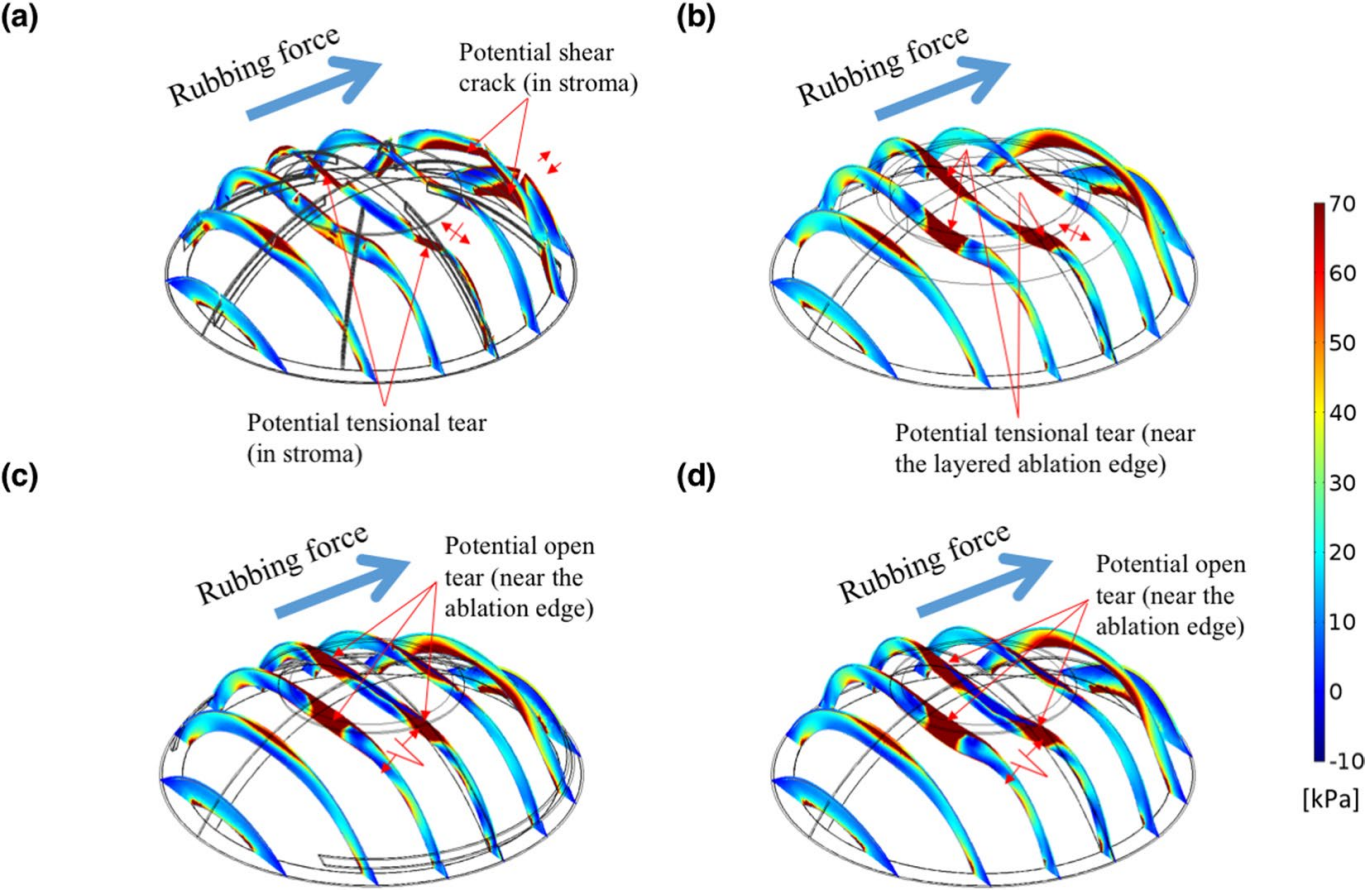
FPS on seven slices of the four models subjected to a certain rubbing force. (**a**) FPS in the RK model, showing the potential fractures occurring at the bottom of the incision with a tensional concentration; (**b**) FPS in the PRK model, showing the zone of the potentially tensional tear surrounding the ablation; (**c**) FPS in the LASIK model and (**d**) FPS in the SMILE model. (**c**,**d**) Indicate the high stresses found around the midperipheral cornea and potentially opening creak zones surrounding the ablation.

The corneal FPS generated by surface traction was applied to simulate these surgery models under rubbing condition. Normal and shear stresses are simply traction vector components, and they are applied to the corneal surface^45^. In the simulation, a normal force of 0.122 N and a shear force of 0.08 N were applied. The area of this traction was 22.9 mm^2^ (5.4 mm in diameter), and the given IOP was 31 mmHg. Figure 4 presents the FPS distribution in seven slice sections of the corneas and visualisations of the deformation in these four models. According to the numerical results, the rubbing force increased the corneal FPS values by more than 2.5 times. High FPS areas were observed in the peripheral cornea in the direction of eye rubbing (right side). For RK, a high FPS resulted in a greater deformation around the peripheral incisional ends, as observed on the right side of the model (Fig. 4a). For PRK, LASIK, and SMILE, the highest FPS areas were found around the ablation and extraction areas because the rubbing force reduced the stresses in the central cornea (Fig. 4b–d). Figures S3,S4 and S5 provide the detailed stress distributions of ${\sigma }_{r}$, ${\sigma }_{\varphi }$, and ${\sigma }_{r\varphi }$, which clarify the crack and tear patterns in terms of opening tear, tensional tear, and shear style, respectively.

### Simulated intraocular pressures for different refractive surgeries

**Figure 5:**
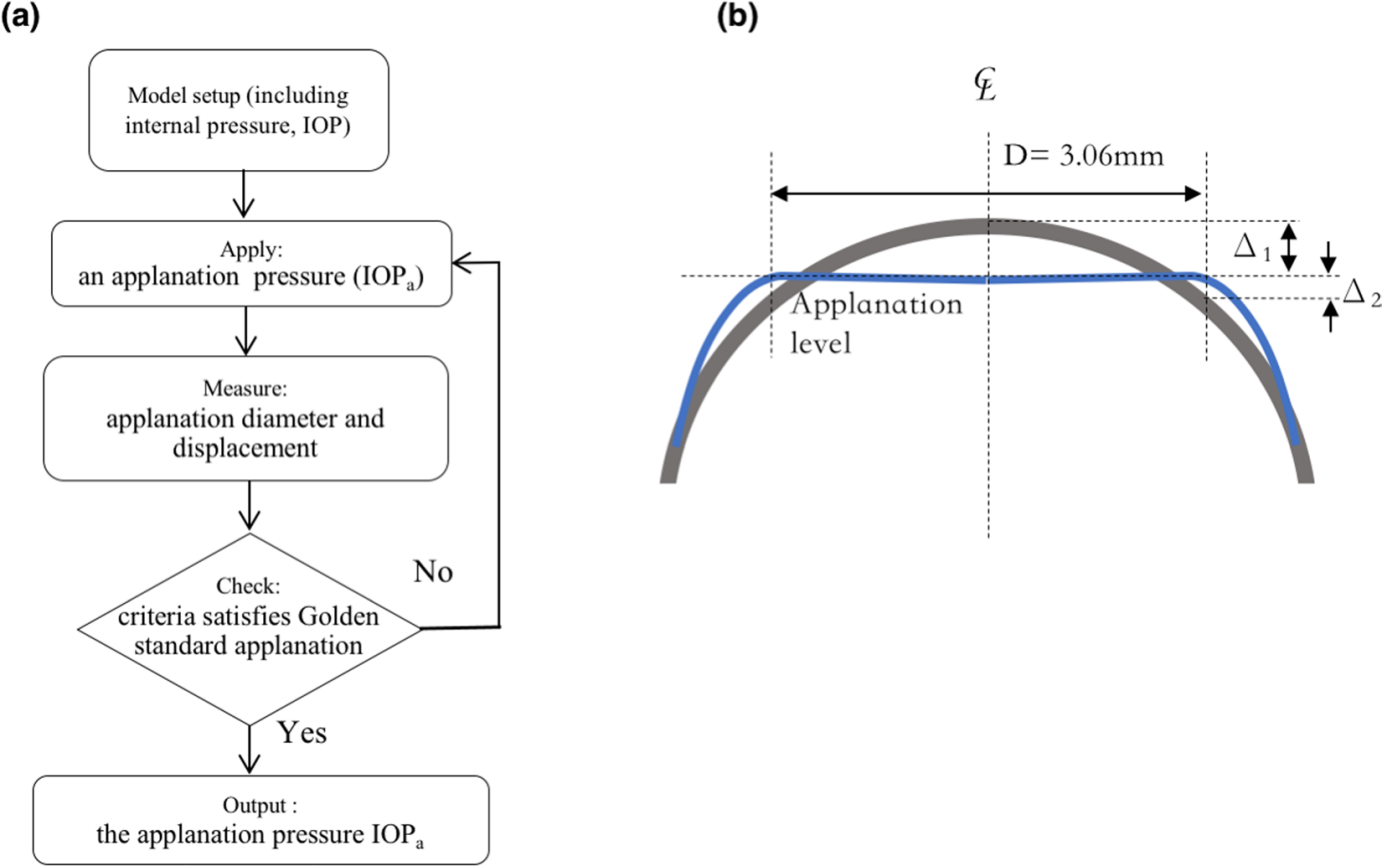
(**a**) Flowchart for the calculation of the IOP_a_ and (**b**) criteria of the applanation in the numerical calculation.

**Figure 6:**
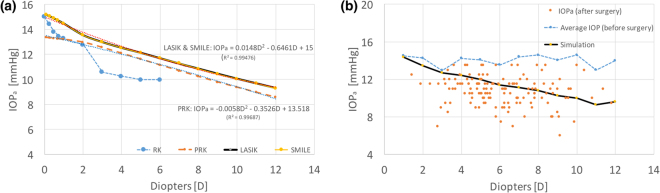
(**a**) Applanation pressure, IOP_a_, associated with myopia corrections of different D levels in the four models at an actual IOP of 15 mmHg and (**b**) applanation pressure, IOP_a_, associated with myopia corrections of different D values from 150 subjects who had undergone LASIK surgery; myopia results were compared to those obtained from simulation with various D levels at the average IOP.

Our models could be applied to estimate the difference between an actual IOP and a measured IOP associated with myopia corrections of different D levels. By definition, the actual IOP equals the applanation pressure (IOP_a_), according to the Goldmann applanation principle^46^. For the correction of postoperative IOP measurement by air-puff or Goldmann tonometer, a general equation depending on the corneal thickness is given: ${{\rm{IOP}}}_{pach}={\rm{IOP}}(550-{\rm{CCT}})\ast 0.05$, where CCT is the central corneal thickness^47^. However, this equation is not appropriate for eyes that have undergone refractive surgical procedures because the uniformity of the corneal thickness and the properties of corneal material are different from those of eyes without surgery. In the current study, we attempted to determine the IOP_a_ based on our FEM models and the Goldmann applanation principle. Figure 5a presents a flowchart for the estimation of the IOP_a_, and the IOP_a_ is adjusted to satisfy the force balance with the IOP in a recursive calculation process^48^. The criterion of the flowchart is that the sum of the scalar displacements at the central point (Δ_1_ downward) and at the edge of the applanation (Δ_2_ upward) must equal the total height measured from this edge of the applanation to the top central point, as shown in Fig. 5b. Figure 6a illustrates the IOP_a_ curves associated with varying D values at an actual IOP (inside) of 15 mmHg. The post-RK cornea was too soft and easily became flattened; hence, the IOP_a_ decreased rapidly and no IOP_a_ could be obtained when myopia of more than 6D was corrected. The trend equation for PRK is IOP_a_ = 13.518–0.3526 * D−0.0058 * D^2^ and the IOP(mmHg)=IOP_a_ + 1.482 + 0.3526 * D + 0.0058 * D^2^. Accordingly, substituting D = 0 into the first equation yielded an IOP_a_ value of 13.518 mmHg; this indicates that PRK reduced the measured IOP by approximately 1.5 mmHg (15–13.518 = 1.482) initially because the remodelling effect of the Bowman’s membrane in PRK broke the stress continuity at the central cornea. The linear term ‘−0.3526*D’ indicates that the decreasing slope is approximately −0.3526 (assuming that ‘0.0058 *D^2^’ is small), and this result is less than 0.6, which is the slope of IOP_*pach*_ (0.05 * 12 μm/D = 0.6/D). The low slope might be attributed to the lack of stiffness of the Bowman’s membrane after PRK. The trends for the LASIK and SMILE models were similar, and the fitting equation is IOP_a_ = 15–0.6461 * D + 0.0148*D^2^. Thus, IOP (mmHg) = IOP_a_ + 0.6461 * D − 0.0148*D^2^. Comparing the intercepts in the LASIK and SMILE models with that in the PRK model, we found that the Bowman’s membrane conforms to a bending rigidity and provides constant and enduring rigidity to oppose the applanation force. We applied the LASIK fitting equation and validated our model with the actual and simulated IOP_a_ values from 150 patients who underwent LASIK surgery, as shown in Fig. 6b; these values agreed with our model. However, we did not have any IOP_a_ data from RK, PRK, and SMILE patients because these cohorts were not present in our hospital.

### Limitations of the FEM models

**Figure 7:**
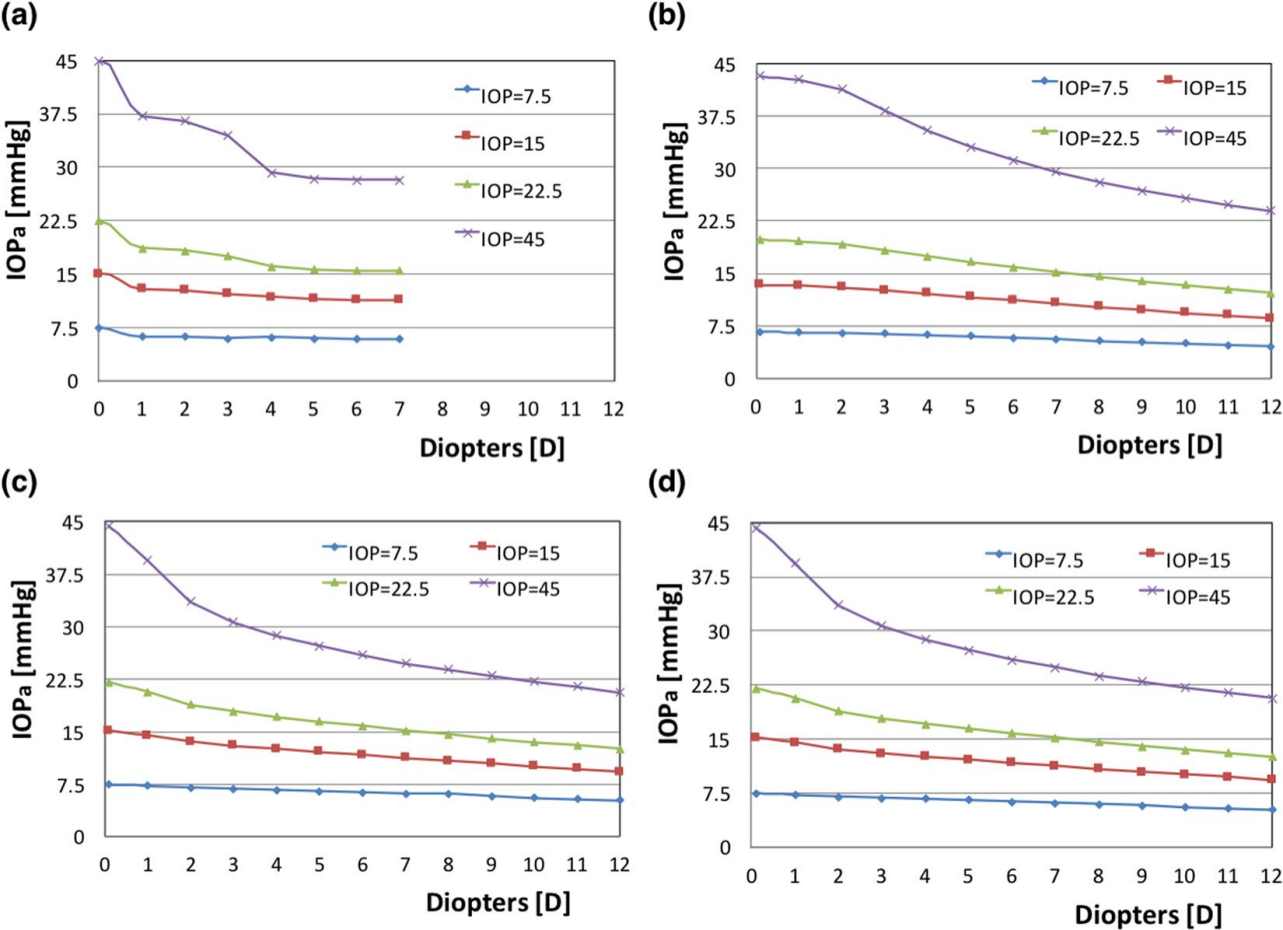
Applanation pressure, IOP_a_, associated with myopia corrections of different D levels at IOPs of 7.5, 15, 22.5, and 45 mmHg: (**a**) RK model, (**b**) PRK model, (**c**) LASIK model, and (**d**) SMILE model.

The equilibrium condition of our models based on the Goldmann’s applanation principle was not satisfied at very low and very high IOPs. Under these two conditions, the observed IOP_a_ became less positively correlated with the actual IOP. Figure 7 shows the relationship between the corrected D and IOP_a_ under various IOP conditions, with the actual IOP values inside being assumed to be 7.5, 15, 22.5, and 45 mmHg. The curve corresponding to the low IOP value (7.5 mmHg) demonstrates a nearly constant IOP_a_ in these models, indicating the rigidity of the structural system providing a considerably greater opposing force than the force obtained from the actual IOP^14^. By contrast, a high IOP could bend the peripheral cornea outwards and flatten the central cornea, resulting in a low IOP_a_ upon applanation. In all models, the three curves associated with the IOP values of 7.5, 15, and 22.5 mmHg reveal similar trends, but in the RK model, the force balance upon applanation cannot be obtained when the myopia correction is greater than 7D. Furthermore, when the IOP was 45 mmHg, the midperipheral cornea deformed more outwardly and the applanation pressure only bent the ablated cornea in the LASIK and the SMILE models. Thus, the soft cornea after ablation had a low IOP_a_, which resulted in a large reduction in the measured IOP. In addition, the outer corneal radius was assumed to be 7.4 mm without applying any IOP, and when the IOP was increased in these models, the eyeball inflated, thus changing the central corneal radius. In the proposed model, we assumed this radial change was too small and could be neglected (approximately 0.295 mm, which was approximately 2% of the diameter; that small radial change had a negligible effect on the stress distribution). However, because of this assumption, the central cornea became slightly steeper (reducing radius), which resulted in an increase of the applanation depth (approximately 28%), as shown in Figure S6a.

## Discussion

To maintain the corneal shape with or without refractive surgical procedures, the Bowman membrane and Descemet’s membrane provide the major bending rigidity, and they resist bending stresses to prevent concave and convex deformations upon applanation, during which the stresses are transferred from the Bowman’s membrane to the Descemet’s membrane before and after applanation. High FPS areas were found around the incisions produced by RK (Figure S2a), and the stress concentrations resulted in small cracks when the applied stress increased. In the PRK model, the stress concentration was at the superficial stroma and the ablated edge of the Bowman’s membrane (Fig. 3b), and the central cornea was displaced outwards, as shown in Fig. 1b. In the LASIK and the SMILE models, the ablations and extraction in the stromal layer did not break the stress continuity in the Bowman’s membrane, and this is advantageous for these models. However, their potential creak zones were at the edge of the ablation around the Bowman’s membrane, as shown in Fig. 3c and d. Tensional stresses were concentrated on the RK incisions around Descemet’s membrane upon eye rubbing. By contrast, the other three models showed stress concentrations near the ablation around the Bowman’s membrane (Fig. 4).

As mentioned, the most crucial structure to maintain the corneal shape and to resist stress is the Bowman’s membrane. For the cornea, we investigated the axial rigidity, which refers to the major resistance of a material against tensile deformation. Mathematically, we could further multiply the Young’s moduli (*E*) with the thicknesses (*h*) of the Bowman’s membrane, stromal layer, and Descemet’s membrane, and the corresponding the axial stiffness (*Eh*) values could be derived to be 5.15, 61.44, and 2.34 [Nt/m], respectively, for a unit width at IOP = 15 and the stromal *E* is estimated by 128 [kPa] (0.3 times of the Bowman’s membrane^28^). These results indicate that the stromal layer contributed the major rigidity (i.e., approximately 89.1% of the axial rigidity). Numerical validation of the inflation test was shown in Figure S7a. We further investigated the bending rigidity, which is the resistance of a material against bending deformation^49^. The area moment of inertia is the geometrical property of an area associated with the rotation of an object with stiffness^50^. We calculated the area moments of inertia (*I* = *bh* ^3^/12 + *bh* * *y* ^2^ where *y* is the distance from the inner side of the cornea and *b* = 1) of the Bowman’s membrane, stromal layer, and Descemet’s membrane, and the calculated values were 2.976 × 10^−12^, 3.97 × 10^−11^, and 5.76 × 10^−16^[m^4^], respectively. The neutral axial was assumed to pass through the inner side of the cornea for the applanation condition. In addition, the bending rigidities (*EI*) were 1.28 × 10^−6^, 5.08 × 10^−6^, and 1.12 × 10^−10^[Nt-m^2^] respectively; these results also indicate that the Bowman’s membrane and Descemet’s membrane, as a pair of forces, provided approximately 20% of the rigidity against bending, despite their extremely small thicknesses. A numerical validation of the bending test is illustrated in Figure S7b. Figure S7a plots the stress-strain curves of our models, which were compatible with Elsheikh’s findings^15^.

**Figure 8:**
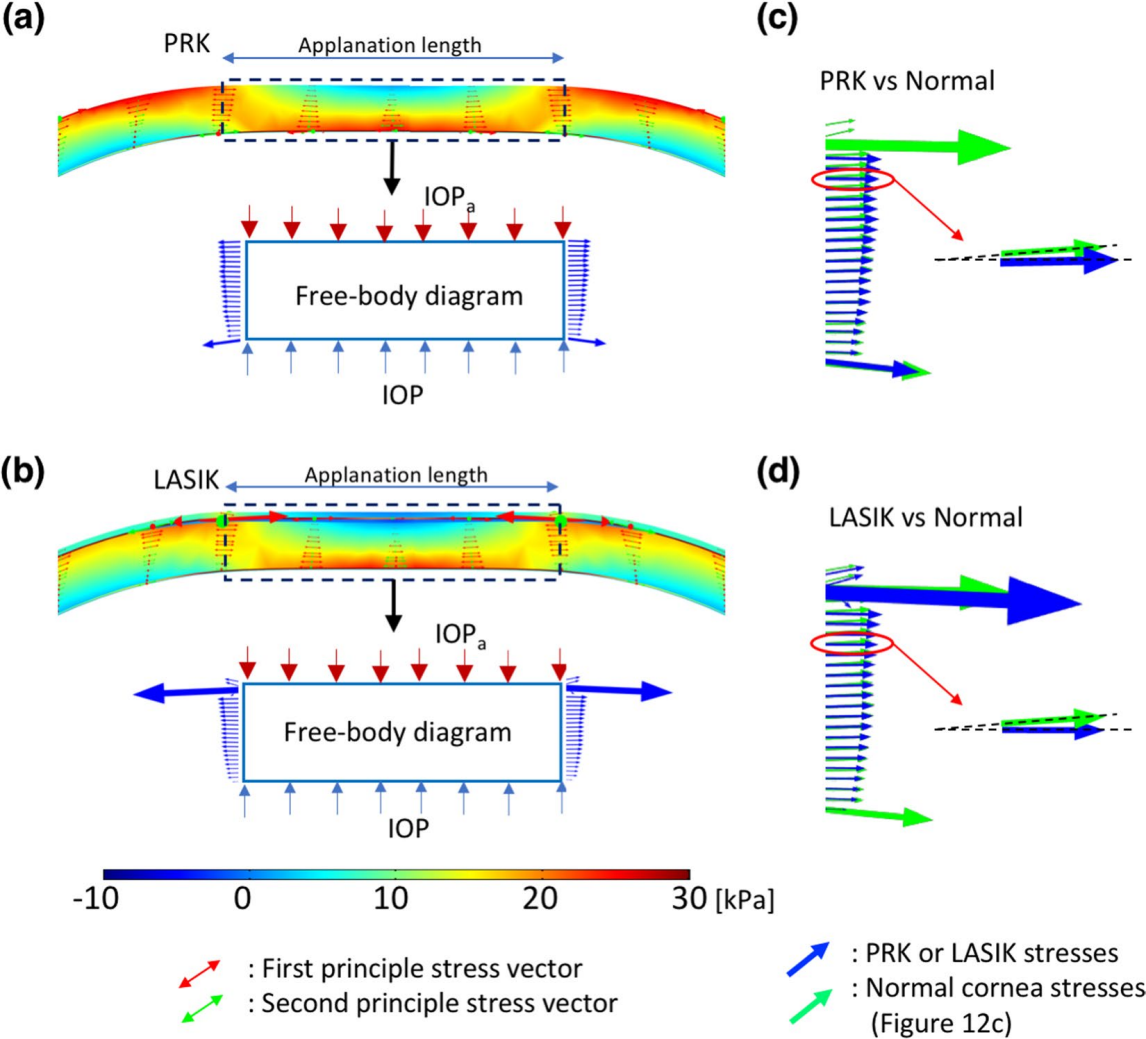
Principal stress directions and free-body diagrams: (**a**) FPS direction of the PRK cornea under applanation; the arrows inside the corneal layers represent the stress directions and values; the inset shows the free-body diagram of the central cornea; (**b**) FPS direction of the LASIK cornea at applanation, and the free-body diagram of the central cornea; (**c**) stress on the cross section of a PRK cornea (blue) and on that of a normal cornea (green, from Fig. 12c); and (**d**) stress on the cross sections of a LASIK (blue) and a normal cornea (green); (**c**,**d**) the stresses are directed obliquely downwards, reducing the IOP_a_ value.

Refractive surgical procedures change the corneal geometry and integrity, break the stress continuity of the corneal structural system, and alter the original equilibrium upon applanation, according to the Goldmann applanation principle. Figure 8 depicts the principle stress directions and free-body diagrams of corneas subjected to PRK and LASIK under applanation conditions; the stresses were obtained from the models in Fig. 2b and c. The free-body diagrams in Fig. 8a and b are given by the black dashed lines; they include the edges of the applanation area. The arrows on these dashed lines indicate the internal stresses and their directions. The changes in internal stress directions and tensile stress directions are fitted to free-body diagrams. These stresses in the cross sections of the free-body diagrams are illustrated by the blue arrows in Fig. 8c and d. Compared with the applanated cornea discussed in next section, the directions of the stresses of the PRK and LASIK models were obliquely downwards and not horizontal. At equilibrium, the vertical components of the stresses contributed the force against the inner pressure (i.e. IOP). Thus, the IOP_a_ was less than the IOP.

**Figure 9:**
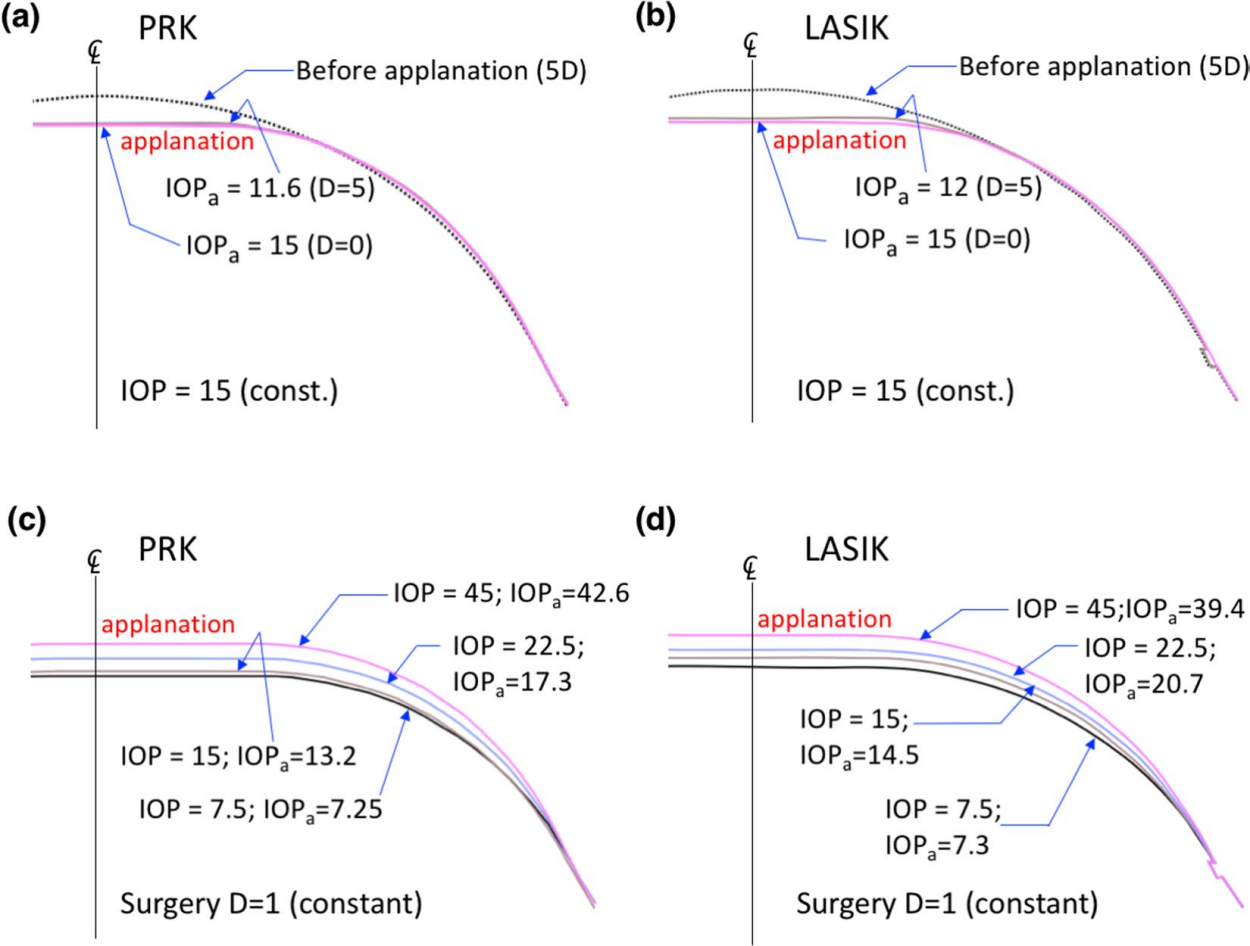
Corneal deformation and flattening upon applanation (**a**) IOP drops from 15 to 11.6 mmHg when the cornea undergone PRK surgery at D = 5; (**b**) IOP drops from 15 to 12 mmHg when the cornea undergone LASIK surgery at D = 5; corneal deformation (D = 1) at applanation with various IOPs for (**c**) the PRK model and for (**d**) the LASIK model.

Figures 9a and b show the deformation and flattening of the central corneas subjected to PRK and LASIK (intended correction of myopia of 0 and 5D) at an IOP value of 15 mmHg. The deformation and bending areas of PRK and LASIK before and after surgery were similar in the central cornea. However, the corneas subjected to surgery required less pressure (IOP_a_ = 11.6 mmHg for PRK and IOP_a_ = 12 mmHg for LASIK) to reach the central flattening level, consistent with Goldmann’s definition. Our FEM simulation results are consistent with the notion that the ablation of the Bowman’s membrane and lenticular extraction of the central cornea did cause the cornea to bend and flatten easily upon applanation. Figure 9c and d reveal deformation and flattening upon applanation in the corneas subjected to PRK and LASIK (1D). Upon applanation, different IOPs induced different bending shapes of the central cornea, wherein a high IOP caused more flattening than did a low IOP. These results indicate that the IOP plays a more crucial role in IOP measurements (IOP_a_) than does the rigidity of the central cornea at low myopia correction by refractive surgery.

In conclusion, our FEM models provide a novel analysis of the stress concentrations by focusing on the stress contribution provided by corneal layers. The results reveal the significance of the Bowman’s membrane in providing bending rigidity and also reveal the potential creak zones in different layers under refractive surgical procedures. The biomechanical viewpoint indicates that redistributed stresses affect IOP values, and the simulation predicts the postoperative IOP in a conditional manner.

## Materials and Methods

### FEM modelling of a normal cornea

**Figure 10:**
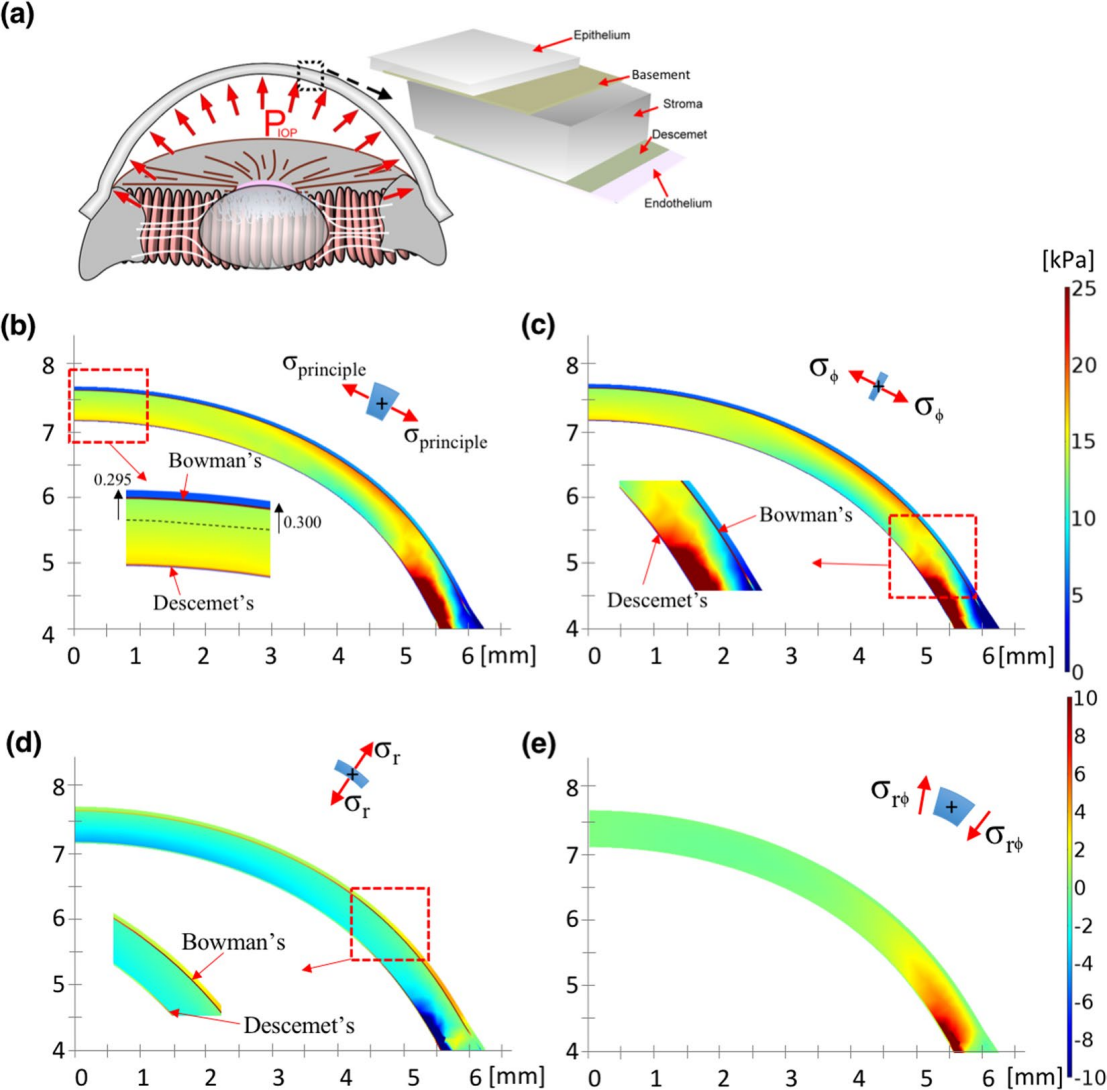
Stress distribution in corneas without applanation. (**a**) Schematic of the six layers of the cornea and the clamped end (limbus and its surrounding tissues); (**b**) FPS distribution on a half slice of the central cornea; (**c**) $\varphi$-direction stress distribution, which describes the tensional stress in the meridian direction and high tension is found in both the Bowman’s membrane and Descemet’s membrane as shown in the inset; (**d**) r-direction stress distribution, which describes the tensional stress in the radial direction and high tension stresses are also found in the Bowman’s membrane and Descemet’s membrane; and (**e**) r-$\varphi$ direction stresses distribution, which describes geometric distortion. High ${\sigma }_{\varphi }$ with very small ${\sigma }_{r\varphi }$ indicates that the cornea only undergoes body expansion with less distortion (Unit: mm and kPa).

In this study, the commercial FEM package Comsol Multiphysics (COMSOL, UK) was used for numerical simulations. A three-dimensional continuum model of the material properties and geometry of corneas, consisting of the six layers of the cornea, namely the epithelium, basement membrane, Bowman’s membrane, stroma, Descemet’s membrane, and endothelium, has been described previously^28,51,52^. Based on these studies, the Young’s modulus of each layer, the hyperelastic and anisotropic material models based on the Holzapfel constitutive model^23,25^, and the N–T and the I–S fibres^12,13,25,30^ were subjected to global analyses (Tables 1 and 2). Among the six layers of a normal cornea, the Bowman’s membrane and Descemet’s membrane have higher Young’s moduli than the other layers do. Following this modelling, we defined the geometry by the outer corneal radius, 7.4 mm, clamped boundary condition (limbus and its surrounding tissues) at the edge^20,27,30^, and liquid pressure on the inner surface with 15 mmHg (indicated as IOP), as depicted in Fig. 10a. The mesh setting of the FEM sequence was of a ‘physically controlled mesh’ type, and the number of nodes was more than those previously reported^13,32,53^. Subsequently, we defined the first principal stress (FPS) distribution on the corneal cross section, where the FPS could be used to evaluate the maximum stress combining the normal and shear stresses^19,54^, as illustrated in Fig. 10b. Based on the data in Fig. 10b, the inner pressure (i.e. IOP) deforms the central cornea outwards by 295 μm which is close to those reported in previous studies^21,37^, and the maximum stress is at the clamped end (limbus and its surrounding tissues) on both sides. Figure 10c–e illustrate the stress in the ϕ-, r-, and rϕ-directions, respectively. Because of the anisotropic properties, stresses applied to the cornea are shown in the N–T cross section. The stress ${\sigma }_{\varphi }$ is the tangential tension and represents the hoop stress stretching parallel to the length of the cornea in the layers; ${\sigma }_{r}$ is the radial stress dominating the body expansion; and ${\sigma }_{r\varphi }$ is a nonaligned stress that pushes one part of a body in one direction and another part of the body in the opposite direction. Accordingly, the levels of ${\sigma }_{\varphi }$ in Bowman’s membrane and Descemet’s membrane are higher than those in the other layers. The cornea can be regarded as a membrane, in which the ratio of thickness to corneal diameter is small and produces little shear stress, except at the clamped end; therefore, the stress ${\sigma }_{r\varphi }$ is very small, as illustrated.

**Table 1:** Geometrical and material properties of the six corneal layers used in the FEM models.

Layers	Thickness (T) [μm]	Density [kg/m^3^]	Poisson’s ratio	Stiffness [kPa] at IOP** [mmHg]			
				7.5	15	22.5	45
Epithelium	53	1.149	0.49	21.3	64.4	108.9	272.3
Basement membrane	2	1.149	0.49	9.7	29.3	49.5	123.8
Bowman’s membrane	12	1.149	0.49	141.9	429	726	1815
Stroma*	480	1.149	0.49	—	—	—	—
Descemet’s	12	1.149	0.49	64.5	195	330	825
Endothelium	5	1.149	0.49	12.9	39	66	165

*Matrix stiffness of stroma is C_1_=5kPa, see Table 2. **Stiffness at zero IOP obtained from study^28^.

**Table 2:** Material parameters for the anisotropic fibrous material of stroma.

*D* [kPa^−1^]	*C*_2_ [kPa]	*K*_1_ [kPa]	*K*_2_	*K*_3_ [kPa]	*K*_4_
13.333	0.0	130	102.643	130	102.643

*Holzapfel’s constitutive model shown in Eq. (2) of ref.^13^ and Eq. (21) of ref.^23^.

### Validation of FEM model by Goldmann applanation principle

**Figure 11:**
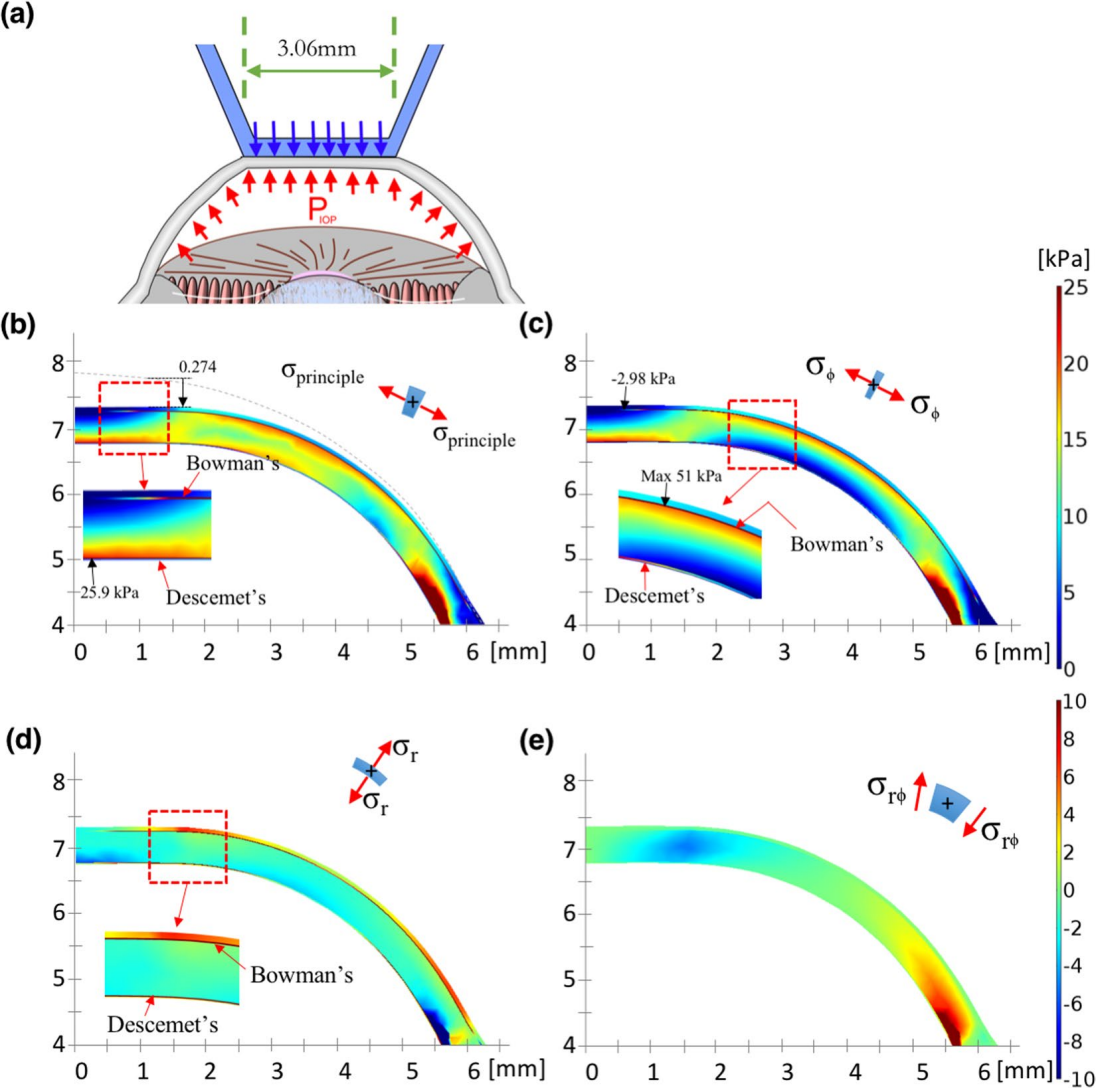
Cornea observed upon applanation. (**a**) Schematic of the Goldmann applanation principle; (**b**) FPS distribution on a half slice of the central cornea; (**c**) $\varphi$-direction stress distribution; (**d**) r-direction stress distribution (the insets in both (**c**) and (**d**) indicate that the applanation pressure induced tension and compression in the Bowman’s membrane, compared with those in Fig. 10b and c); and (**e**) low r-$\varphi$ shear stresses, indicating that the corneas had more body expansion (tension area) and contraction (compression area) during applanation (Unit: mm and kPa).

**Figure 12:**
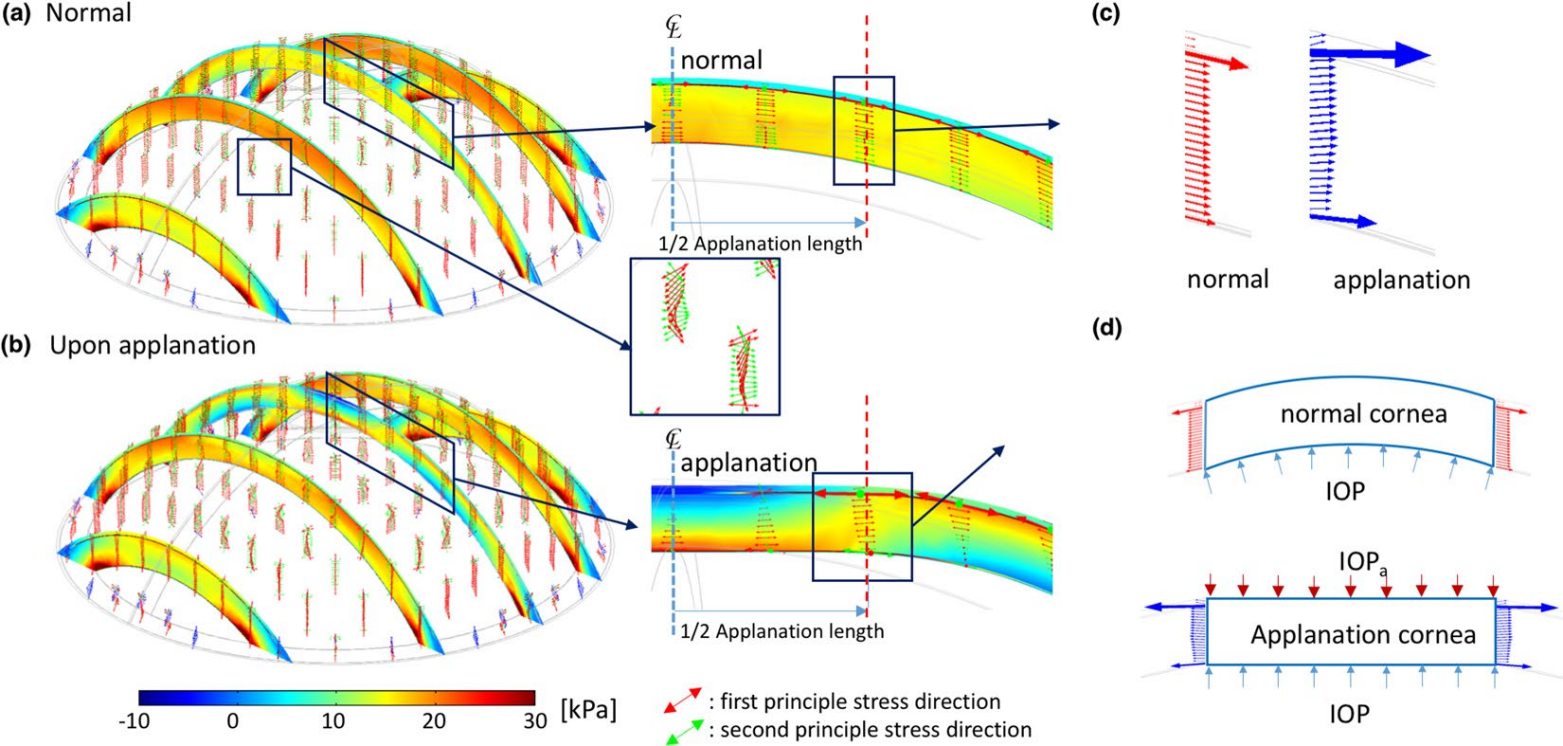
Principal stress directions and their free-body diagrams. (**a**) and (**b**) FPS direction of a normal cornea obtained from the central cornea of Fig. 10b and b without and with applanation. The arrows inside the corneal layers represent the stress directions and values (their length). The red and dashed lines represent the edge of the applanation, and it will be the right boundary of the free-body diagram. (**c**) The distribution of these stress arrows along the lines becomes the stress distribution on the cross section of the free-body diagram. (**d**) Free-body diagram of the central cornea with 3.06-mm diameter. The stress distribution along the left cross section is mirrored from the right side because the cornea is axial symmetric. The contribution of the vertical component of the stresses (red arrows) of without applanation balances the vertical components of the IOP stress. The direction of the stresses (blue arrows) of the applanated cornea is almost horizontal; thus, IOP_a_ stresses and IOP stresses balance each other.

We also applied the Goldmann applanation simulation to validate our FEM model, as described previously^21,39^. According to the principle of Goldmann tonometry, the opposing forces from corneal rigidity and the tear film are equal to and in equilibrium with the IOP, which is also a uniform pressure, to be determined from the applanation force required to flattened an area of the cornea with a 3.06-mm diameter^46^. The equilibrium between the IOP and the outer applanation pressure was thus used as a criterion to validate our FEM model, as depicted in Fig. 11a. Accordingly, Fig. 11b illustrates the FPS distribution and the flattened surface upon applanation. When the forces were fitted in Elsheikh’s studies, the stress and displacement at the central cornea were 25.9 kPa and 274 μm; those theoretical predictions were close to their experimental results, namely 25 kPa and 253 μm^36,39^. Figure 11c illustrates the stress ${\sigma }_{\varphi }$, indicating that the stress values become negative (−2.98 kPa) in the central top cornea (compression) and positive in the central bottom cornea (tension); this nonuniform stress represents the bending stress inside these layers. Moreover, the maximum tension is in the peripheral part of the mid-upper cornea. Figure 11d depicts the stress, ${\sigma }_{r}$, indicating that the stress values change from negative (upper-peripheral cornea, force outwards) to positive (bottom cornea, force inwards). Figure 11e shows the small shear stresses in cornea. We also present free-body diagrams^55^ of the applanated cornea to explain the equilibrium of the applied forces, as shown in Fig. 12. Figure 12a and b illustrate the two principal stresses and the directions of the normal and the applanated corneas. The inset figures depict the stresses in slices, in which the arrows indicate the stress directions. Figure 12c depicts the stresses, arising from the principal stresses illustrated in the insets of Fig. 12a and b. The cross section of the cornea is 1.53 mm in radius; the directions of tensile stresses in the normal cornea are oriented obliquely but those in the applanated cornea are mostly horizontal. Figure 12d shows free-body diagrams; within the applanation diameter (3.06 mm), the stress vectors in the normal cornea are oblique to the peripheral cornea to counterbalance the IOP. Upon applanation, the stress vectors are extended horizontally when the applanation pressure, IOP_a_, is equal to the IOP.

### FEM model for refractive surgery

**Figure 13:**
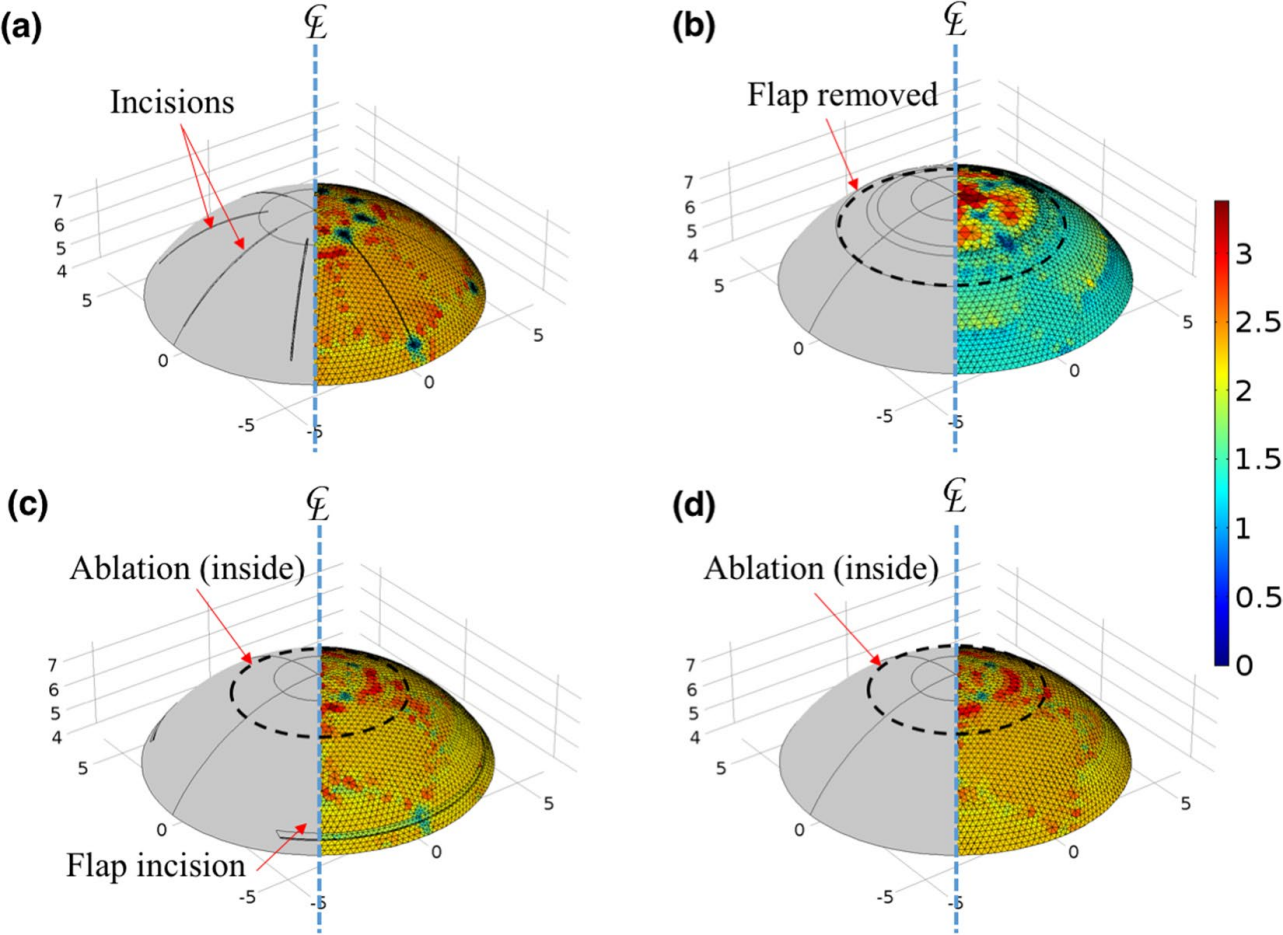
Simulation models (left) and their mesh volume (right), and the colour legend shows the scale of the mesh size. (**a**) RK, (**b**) PRK, (**c**) LASIK, and (**d**) SMILE (ablations inside the dashed line. Unit: 1 × 10^−4^ mm^3^).

In the current study, we applied our FEM model to four types of refractive surgery. For RK, four, six, or eight radial incisions were assumed to be made with a diamond knife at 90% corneal depth, with the optical zones ranging from 4.5 to 3.0 mm; these incisions were simulated to correct myopia from 1.0 to 7.0 D, as previously described^56,57^. Because the incisions were not healed and filled with corneal epithelial cells, the rigidity of the cornea undergoing RK were low^28,58^. The FEM model for RK is depicted in Fig. 13a. For PRK, parts of the stroma and Bowman’s membrane were ablated to treat myopia between 1.0 and 12.0 D in the simulation^59,60^. A 50-μm thick epithelial flap was removed; the ablation depth for stromal tissue was 12 μm/D, and the ablation zone had a diameter of 6.0 mm. The FEM model for PRK is illustrated in Fig. 13b. For LASIK, the first step involved creating a corneal flap, where a microkeratome or femtosecond laser is used to cut through the epithelium and Bowman’s membrane to the stroma to a 130–160-μm depth. The exposed corneal stroma was then reshaped using an excimer laser beam to correct myopia. The corneal flap was then placed in its original position, where it self-sealed and acted as a natural bandage^61^. In the simulation, the thickness of the flap was 160 μm; the ablation depth was 12 μm/D, with the ablation zone having a diameter of 5.0 mm. Here, the corneal flap was assumed to have sealed spontaneously in the stromal layer, but the periphery of the flap remained unsealed. The FEM model for LASIK is depicted in Fig. 13c. Finally, for SMILE, the layers of the lenticule were outlined and disrupted using a femtosecond laser, after which the lenticule was extracted using a stripper through a small corneal incision also created using a femtosecond laser^62^. In the simulation, the thickness of the 160-μm superficial stromal layer was not ablated with a femtosecond laser. The diameter of the leticule was 5.0 mm, and the thickness of the extracted lenticule was 12 μm/D. The FEM model for SMILE is depicted in Fig. 13d. In our models, the meshes were composed of more than 0.138 million quadratic full-integration mixed-formulation solid elements. Different degrees of freedom were used to perform the simulations, and the changes of the apical displacements and the apical FPS for different mesh densities tested the quality of these meshes. The results demonstrated that the models were adequate with the aforementioned degrees of freedom (828,578, 717,906, 642,852, 464,395); the relative change of the apical displacement was less than 1.93% and the change of the apical FPS was less than 0.06% as shown on Table 3.

**Table 3:** Mesh sensitivity analysis for the changes in the apical displacements and the apical FPS relative to numbers of degrees of freedom.

RK	Number of degree of freedom	413,432	683,791	**828,576**	1,266,756
	Linear error	1.10E-09	1.10E-09	6.90E-10	6.00E-10
	Relative cange in apical disp.	—	3.27%	1.78%	**1.93%**
	Relative cange in apical FPS	—	4.40%	0.77%	0.05%
PRK	Number of degree of freedom	231,494	363,701	**717,906**	1,009,708
	Linear error	5.80E-10	4.50E-10	3.90E-10	2.60E-10
	Relative cange in apical disp.	—	0.02%	0.02%	0.08%
	Relative cange in apical FPS	—	0.04%	0.06%	**0.06%**
LASIK	Number of degree of freedom	309,585	464,395	**642,852**	1,013,765
	Linear error	8.90E-10	6.30E-10	2.80E-10	2.90E-10
	Relative cange in apical disp.	—	0.03%	0.00%	0.10%
	Relative cange in apical FPS	—	0.51%	0.10%	0.05%
SMILE	Number of degree of freedom	253,242	405,396	**464,395**	995,119
	Linear error	7.60E-10	3.70E-10	6.30E-10	3.10E-10
	Relative cange in apical disp.	—	0.17%	0.10%	0.10%
	Relative cange in apical FPS	—	0.17%	0.19%	0.02%

## Electronic supplementary material

Supplementary figures

## References

[1] Mysore N, Krueger R (2015). Advances in Refractive Surgery: May 2013 to June 2014. Asia Pac J Ophthalmol (Phila).

[2] Santhiago MR, Giacomin NT, Smadja D, Bechara SJ (2016). Ectasia risk factors in refractive surgery. Clin Ophthalmol.

[3] Parker P (1994). Overview of refractive surgery. J Ophthalmic Nurs Technol.

[4] Randleman JB, Russell B, Ward MA, Thompson KP, Stulting RD (2003). Risk factors and prognosis for corneal ectasia after LASIK. Ophthalmology.

[5] Rabinowitz YS (2006). Ectasia after laser *in situ* keratomileusis. Curr Opin Ophthalmol.

[6] Binder PS (2005). Keratoconus and corneal ectasia after LASIK. J Refract Surg.

[7] Pinero DP, Alcon N (2015). Corneal biomechanics: a review. Clin Exp Optom.

[8] Vellara HR, Patel DV (2015). Biomechanical properties of the keratoconic cornea: a review. Clin Exp Optom.

[9] Saragoussi JJ (1990). Radial keratotomy. Fortschr Ophthalmol.

[10] Kwon TH, Ghaboussi J, Pecknold DA, Hashash YMA (2010). Role of Corneal Biomechanical Properties in Applanation Tonometry Measurements. J Refract Surg.

[11] Montes-Mico R, Charman WN (2001). Intraocular pressure after excimer laser myopic refractive surgery. Ophthalmic Physiol Opt.

[12] Ariza-Gracia MA, Zurita J, Pinero DP, Calvo B, Rodriguez-Matas JF (2016). Automatized Patient-Specific Methodology for Numerical Determination of Biomechanical Corneal Response. Ann Biomed Eng.

[13] Ariza-Gracia MA, Zurita JF, Pinero DP, Rodriguez-Matas JF, Calvo B (2015). Coupled biomechanical response of the cornea assessed by non-contact tonometry. A simulation study. PLoS One.

[14] Ariza-Graciaa MA, Ortillésa A, Cristóbala JA, Rodríguez Matase JF, Calvoa B (2017). A numerical-experimental protocol to characterize corneal tissue with an application to predict astigmatic keratotomy surgery. J Mech Behav Biomed.

[15] Elsheikh A, Alhasso D, Rama P (2008). Biomechanical properties of human and porcine corneas. Experimental Eye Research.

[16] Elsheikh A, McMonnies CW, Whitford C, Boneham GC (2015). *In vivo* study of corneal responses to increased intraocular pressure loading. Eye Vis (Lond).

[17] Whitford C (2016). *Ex vivo* testing of intact eye globes under inflation conditions to determine regional variation of mechanical stiffness. Eye Vis (Lond).

[18] Joda AA, Shervin MM, Kook D, Elsheikh A (2016). Development and validation of a correction equation for Corvis tonometry. Comput Methods Biomech Biomed Engin.

[19] Nejad TM, Foster C, Gongal D (2014). Finite element modelling of cornea mechanics: a review. Arq Bras Oftalmol.

[20] Elsheikh A (2010). Finite element modeling of corneal biomechanical behavior. J Refract Surg.

[21] Elsheikh A, Wang D (2007). Numerical modelling of corneal biomechanical behaviour. Comput Methods Biomech Biomed Engin.

[22] Roy AS, Dupps WJ (2011). Patient-specific modeling of corneal refractive surgery outcomes and inverse estimation of elastic property changes. J Biomech Eng.

[23] Alastrue V, Calvo B, Pena E, Doblare M (2006). Biomechanical modeling of refractive corneal surgery. J Biomech Eng.

[24] Studer HP (2015). Biomechanical Modeling of Femtosecond Laser Keyhole endokeratophakia Surgery. J Refract Surg.

[25] Lanchares E, Calvo B, Cristobal JA, Doblare M (2008). Finite element simulation of arcuates for astigmatism correction. J Biomech.

[26] Pandolfi A, Holzapfel GA (2008). Three-dimensional modeling and computational analysis of the human cornea considering distributed collagen fibril orientations. J Biomech Eng.

[27] Bryant MR, McDonnell PJ (1996). Constitutive laws for biomechanical modeling of refractive surgery. J Biomech Eng.

[28] Last JA, Thomasy SM, Croasdale CR, Russell P, Murphy CJ (2012). Compliance profile of the human cornea as measured by atomic force microscopy. Micron.

[29] Bryant MR, Velinsky SA (1991). Design of Keratorefractive Surgical-Procedures - Radial Keratotomy. J Mech Design.

[30] Whitford C, Studer H, Boote C, Meek KM, Elsheikh A (2015). Biomechanical model of the human cornea: considering shear stiffness and regional variation of collagen anisotropy and density. J Mech Behav Biomed Mater.

[31] Cheng X, Petsche SJ, Pinsky PM (2015). A structural model for the *in vivo* human cornea including collagen-swelling interaction. J R Soc Interface.

[32] Pinsky PM, Datye DV (1991). A microstructurally-based finite element model of the incised human cornea. J Biomech.

[33] Su P, Yang Y, Zhang LY, Huang L (2016). Biomechanical simulation of needle insertion into cornea based on distortion energy failure criterion. Acta Bioeng Biomech.

[34] Ford, M. R., Dupps, W. J., Rollins, A. M., Roy, A. S. & Hu, Z. L. Method for optical coherence elastography of the cornea. *J Biomed Opt* **16** (2011). 10.1117/1.3526701 PMC3041813 21280911

[35] Sinha Roy A, Dupps WJ, Roberts CJ (2014). Comparison of biomechanical effects of small-incision lenticule extraction and laser *in situ* keratomileusis: finite-element analysis. J Cataract Refract Surg.

[36] Elsheikh A, Wang D, Kotecha A, Brown M, Garway-Heath D (2006). Evaluation of Goldmann applanation tonometry using a nonlinear finite element ocular model. Ann Biomed Eng.

[37] Anderson K, El-Sheikh A, Newson T (2004). Application of structural analysis to the mechanical behaviour of the cornea. J R Soc Interface.

[38] Lanchares E, Calvo B, del Buey MA, Cristobal JA, Doblare M (2010). The Effect of Intraocular Pressure on the Outcome of Myopic Photorefractive Keratectomy: A Numerical Approach. J Healthc Eng.

[39] Elsheikh A, Alhasso D, Gunvant P, Garway-Heath D (2011). Multiparameter correction equation for Goldmann applanation tonometry. Optom Vis Sci.

[40] Kamma-Lorger CS (2010). Collagen and mature elastic fibre organisation as a function of depth in the human cornea and limbus. J Struct Biol.

[41] Meek KM, Blamires T, Elliott GF, Gyi TJ, Nave C (1987). The organisation of collagen fibrils in the human corneal stroma: a synchrotron X-ray diffraction study. Curr Eye Res.

[42] Aghamohammadzadeh H, Newton RH, Meek KM (2004). X-ray scattering used to map the preferred collagen orientation in the human cornea and limbus. Structure.

[43] Abahussin M (2009). 3D collagen orientation study of the human cornea using X-ray diffraction and femtosecond laser technology. Invest Ophthalmol Vis Sci.

[44] Delalleau A, Josse G, Lagarde JM, Zahouani H, Bergheau JM (2006). Characterization of the mechanical properties of skin by inverse analysis combined with the indentation test. J Biomech.

[45] Elsheikh A, Ross S, Alhasso D, Rama P (2009). Numerical study of the effect of corneal layered structure on ocular biomechanics. Curr Eye Res.

[46] Okafor KC, Brandt JD (2015). Measuring intraocular pressure. Curr Opin Ophthalmol.

[47] Kohlhaas M (2006). Effect of central corneal thickness, corneal curvature, and axial length on applanation tonometry. Arch Ophthalmol.

[48] Rachanski, V. Calculation of Recursive Programs. *Dopov Akad Nauk A*, 853–855 (1979).

[49] Diggins PM, Hu MY, Deserno M (2014). Buckling Gel-Phase Membranes is a Way to Measure their Mean Bending Regidity. Biophys J.

[50] Cherng JG, Chen XF (1996). Potentials and limitations of acoustic metrology for dimensional conformance: A comparative study. J Sound Vib.

[51] Guarnieri, F. A. *Corneal Biomechanics and Refractive Surgery*. VII edn (Springer International Publishing AG, 2015).

[52] Bojtár ZBI (2013). Biomechanical modelling of the accommodation problem of human eye. periodica polytechnica Civil Engineering.

[53] Cheng, X., Petsche, S. J. & Pinsky, P. M. A structural model for the *in vivo* human cornea including collagen-swelling interaction. *J R Soc Interface* **12** (2015). 10.1098/rsif.2015.0241 PMC4535401 26156299

[54] Elsheikh A (2010). Finite Element Modeling of Corneal Biomechanical Behavior. J Refract Surg.

[55] Puri A (1996). The art of free-body diagrams. Physics Education.

[56] Ellis, W. *A textbook of radial keratotomy and astigmatism surgery*. 2 edn (1986).

[57] Waring GO (1983). Rationale for and design of the National Eye Institute Prospective Evaluation of Radial Keratotomy (PERK) Study. Ophthalmology.

[58] Bergmanson J, Farmer E, Goosey J (2001). Epithelial plugs in radial keratotomy: the origin of incisional keratitis?. Cornea.

[59] Bohm A (1997). Biomechanical investigation of corneal stability after PRK. Ophthalmologe.

[60] McDonnell PJ, Moreira H, Clapham TN, D’Arcy J, Munnerlyn CR (1991). Photorefractive keratectomy for astigmatism. Initial clinical results. Arch Ophthalmol.

[61] Bower KS, Weichel ED, Kim TJ (2001). Overview of refractive surgery. Am Fam Physician.

[62] Reinstein DZ, Archer TJ, Gobbe M (2014). Small incision lenticule extraction (SMILE) history, fundamentals of a new refractive surgery technique and clinical outcomes. Eye Vis (Lond).

