# Supplementary material for: Biomechanical Simulation of Stress Concentration and Intraocular Pressure in Corneas Subjected to Myopic Refractive Surgical Procedures
Source: Sci Rep. 2017 Oct 24;7:13906. doi: 10.1038/s41598-017-14293-0 (PMC5655007; doi:10.1038/s41598-017-14293-0)
Supplement: Supplementary file 1 — Supplementary figures [file 41598_2017_14293_MOESM1_ESM.pdf]

Biomechanical Simulation of Stress Concentration and Intraocular Pressure in Corneas Subjected to Myopic Refractive Surgical Procedures

Po-Jen Shih[1], I-Jong Wang[2], Wen-Feng Cai[1] & Jia-Yush Yen[3]

[1]Department of Civil and Environmental Engineering, National University of Kaohsiung, 81148, Kaohsiung, Taiwan.

[2]Department of Ophthalmology, College of Medicine, National Taiwan University, 10048, Taipei, Taiwan.

[3]Department of Mechanic Engineering, National Taiwan University, 10617, Taipei, Taiwan.

Correspondence and requests for materials should be addressed to I-J. Wang (ijong@ms8.hinet.net)

Supplemental figures

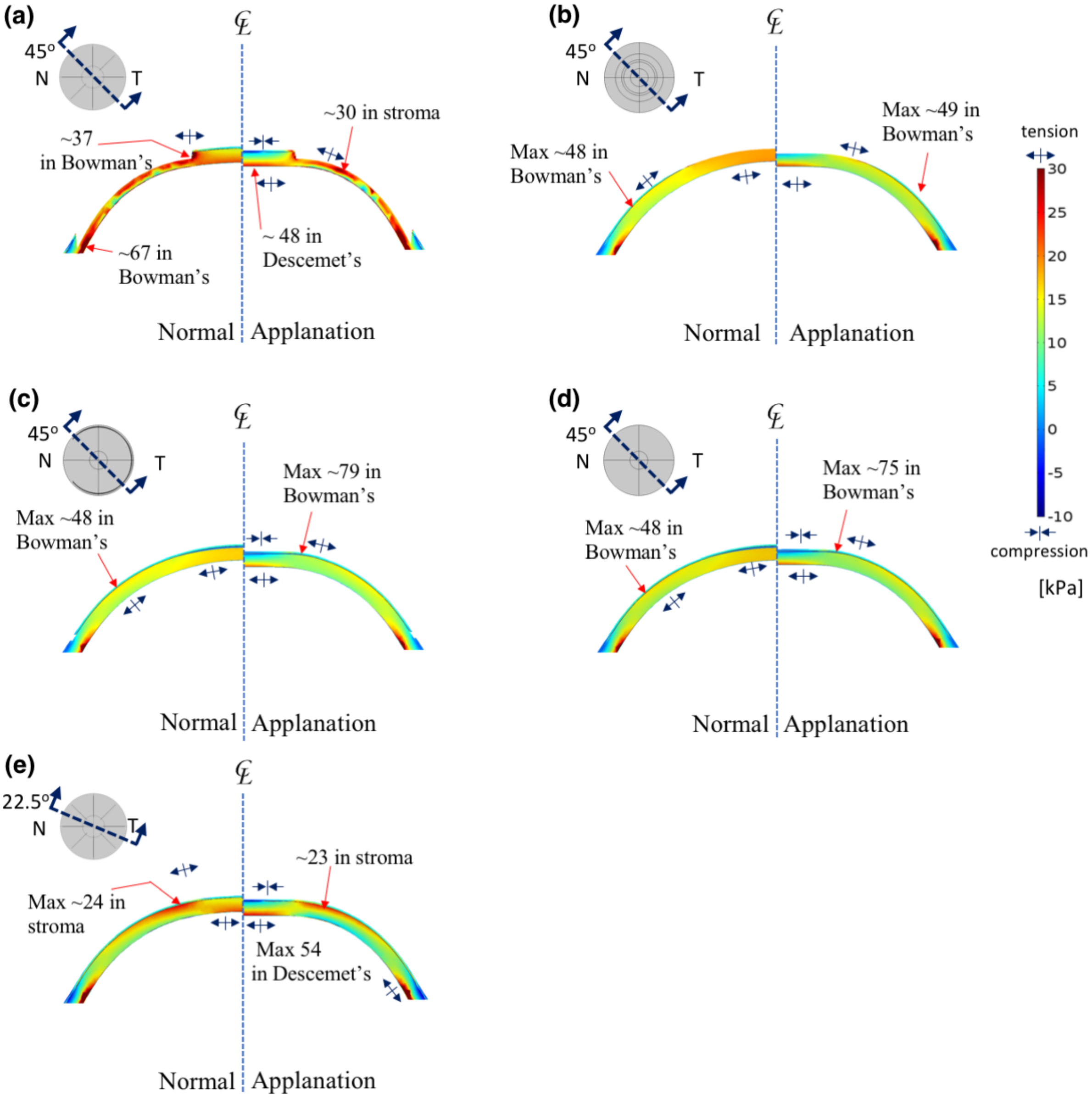

Figure S1. Hoop stress distribution, σ_{ϕ}, which represents circumferential tension in the tangential direction along the cornea, on the 45° slices of the corneas of the four models without (left) and with (right) applanation: (a) RK: the potential creak zone is near the middle periphery of the incision; (b) PRK: the highest stress is found near the edge of the ablation; (c) LASIK: the highest stress is around the edge of ablation; (d) SMILE, the highest stress zone is the same as that of LASIK. The stresses in PRK, LASIK, and SMILE are less than those in Figure 2, which indicates the 45° axes of the fibres contribute less strength. (e) RK on 22.5° slice; the high stress is similar to Figure 2a at the central bottom (value of the legend shows tension; unit: kPa)

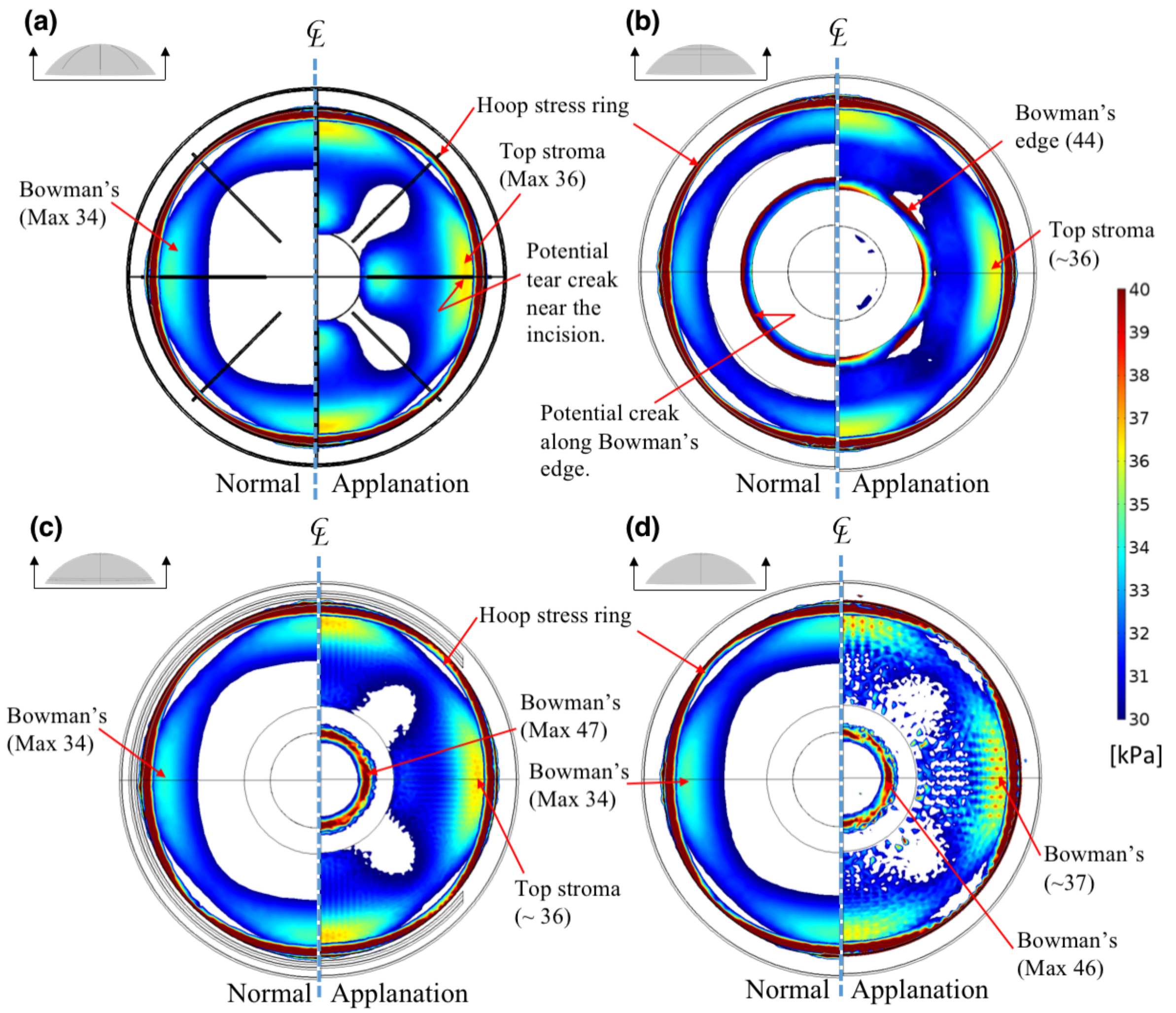

Figure S2. High FPS areas (bottom view showing FPS > 30 kPa) of the four models under without (left) and with (right) applanation. (a) RK: the potential creak zone is near the bottom of the incision; (b) PRK: the highest stresses are near the edges of the ablation and in N–T or I–S directions; (c) LASIK: the potential creak zone is near the edge of ablation; (d) SMILE: the potential creak zone is the same as LASIK but the stress is lower that of LASIK (unit: kPa)

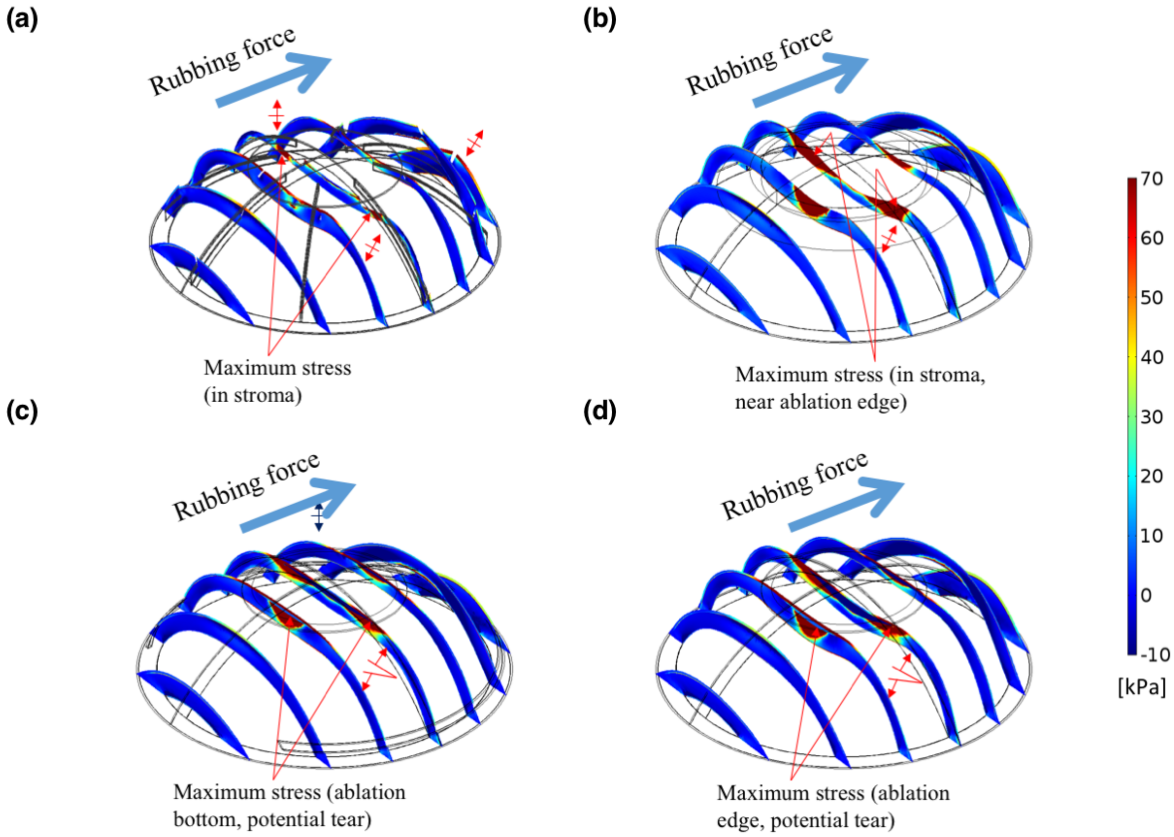

Figure S3. Hoop stress distribution, σ_r, which represents radial tension on seven slices of the four models subjected to a certain rubbing force. (a) RK model; (b) PRK model; (c) LASIK model; and (d) SMILE model. The highest stresses around the peripheral cornea indicate the opening force in radial directions

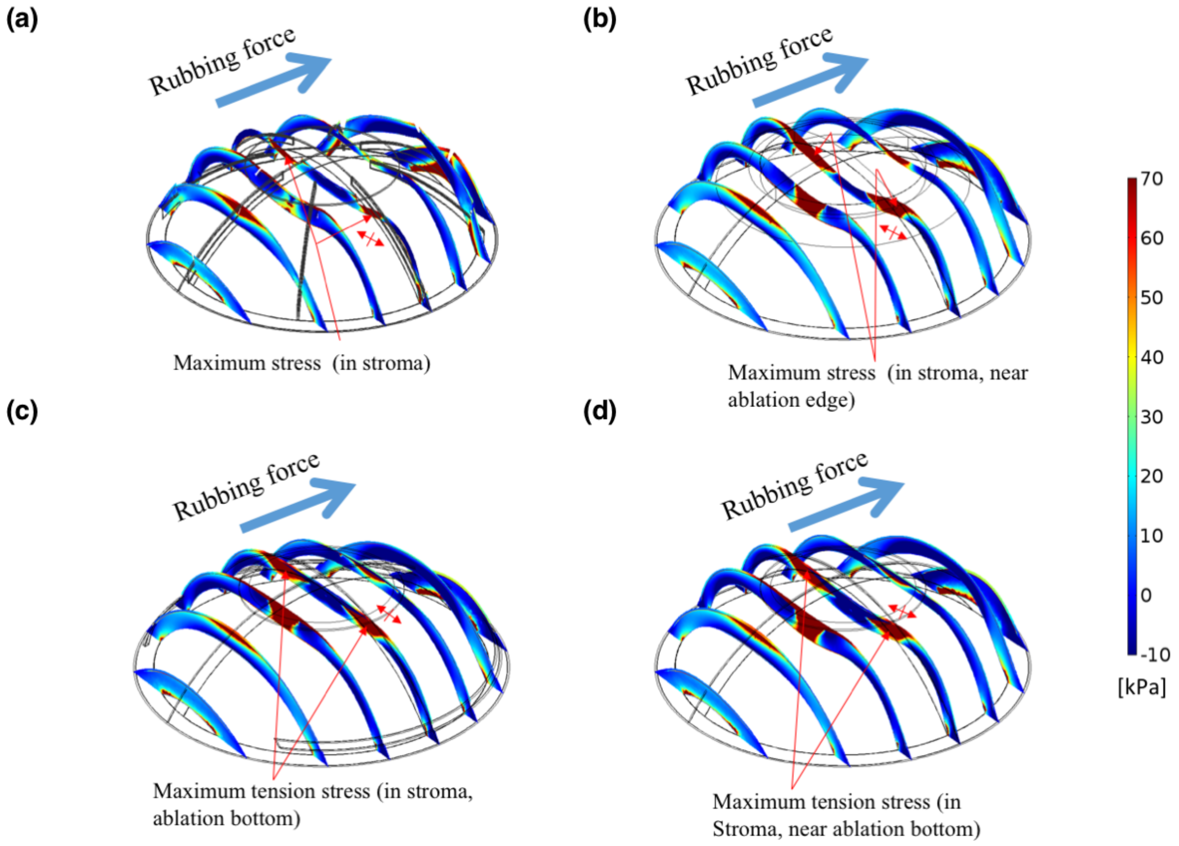

Figure S4. Hoop stress distribution, σ_ϕ, which represents tangential tension on seven slices of the four models subjected to a certain rubbing force. (a) RK model; (b) PRK model; (c) LASIK model; and (d) SMILE model. The highest stresses around the peripheral cornea are caused by the tensional force in meridian direction

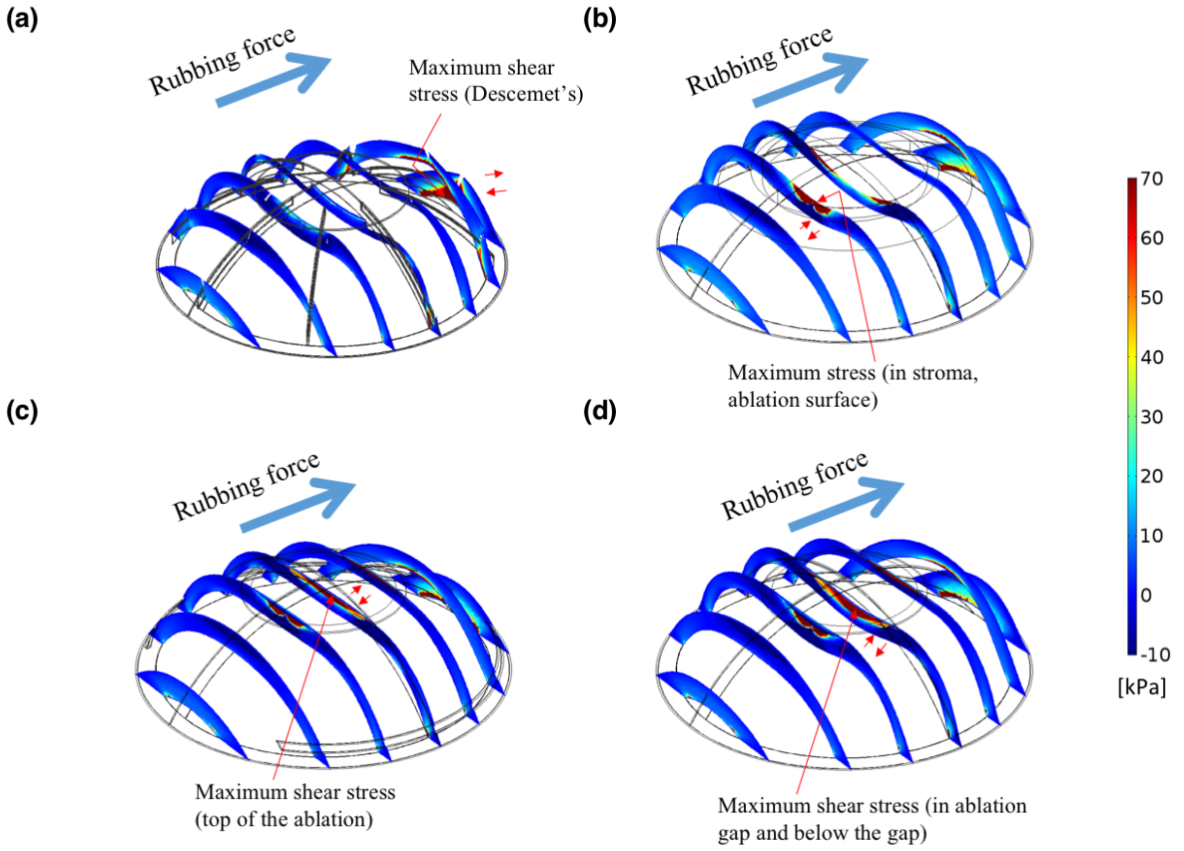

Figure S5. Hoop stress distribution, $\sigma_{r\phi}$, which represents shear stress on seven slices of the four models subjected to a certain rubbing force. (a) RK model; (b) PRK model; (c) LASIK model; and (d) SMILE model. The highest stresses are the shear stresses inside the cornea

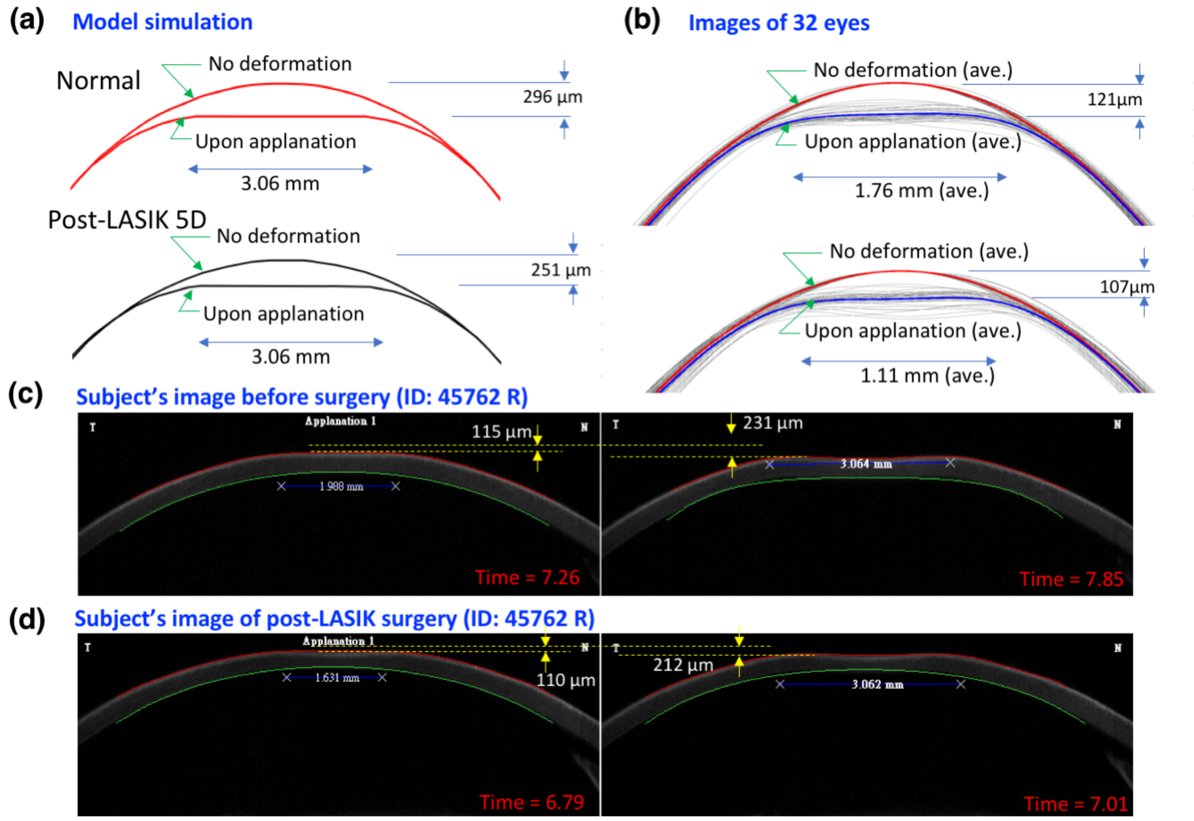

Figure S6. Validation of the corneal deformation. (a) Geometric shapes of the central cornea before and upon applanation in a normal cornea and a cornea that has undergone LASIK surgery. The applanation length is controlled at 3.06 mm; (b) Corneal images recorded by Corvis® ST before and upon applanation in normal and post-LASIK surgery conditions. Grey curves are the deformed shapes recorded from 32 subjects; the red curves are the average shapes before applanation, and the blue curves are the average shapes after applanation. The average applanation lengths are 1.76 and 1.11 mm, much less than 3.06 mm; (c) corneal images of a normal subject and (d) corneal images derived from postsurgery cornea at Corvis' first applanation and at 3.06 applanation length. Comparison of (a), (c), and (d) shows the simulated deflections are 24% and 5.2% larger than that of the subject's cornea in pre- and post-LASIK conditions, and that may be due to geometric problem and material properties

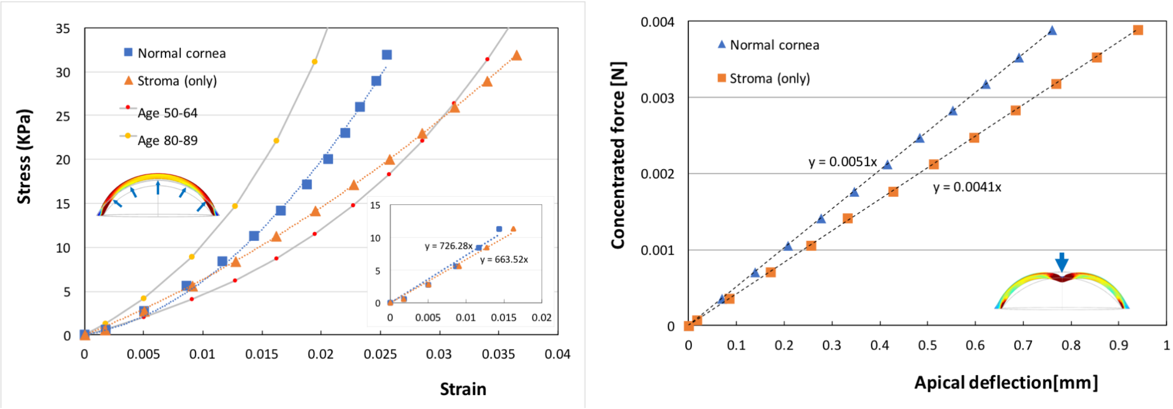

Figure S7. The tensional and bending rigidities of the whole cornea and the stroma. (a) A posterior pressure was applied to extend the cornea, and the apical displacements were measured with the pressures. Then their relation was transferred to the strain–stress relationship, which satisfied the human range[15]. In the inset, the slopes of the two curves with small deformation are 726.28 (normal) and 663.52 (stroma), and thus the tensional rigidity of stroma occupies 91.35% of the total. (b) A concentrated force applied at the central cornea bends the cornea, and the apical deflection indicates the bending rigidity of the corneal structure. The slopes of the two lines are 0.0051 (normal) and 0.0041 (stroma), and thus the bending rigidity of stroma occupies 80.4% of the total
